# Heterogeneous susceptibility to rotavirus infection and gastroenteritis in two birth cohort studies: Parameter estimation and epidemiological implications

**DOI:** 10.1371/journal.pcbi.1007014

**Published:** 2019-07-26

**Authors:** Joseph A. Lewnard, Benjamin A. Lopman, Umesh D. Parashar, Aisleen Bennett, Naor Bar-Zeev, Nigel A. Cunliffe, Prasanna Samuel, M. Lourdes Guerrero, Guillermo Ruiz-Palacios, Gagandeep Kang, Virginia E. Pitzer

**Affiliations:** 1 Division of Epidemiology and Biostatistics, School of Public Health, University of California, Berkeley, Berkeley, California, United States of America; 2 Department of Epidemiology, Rollins School of Public Health, Emory University, Atlanta, Georgia, United States of America; 3 Division of Viral Diseases, Centers for Disease Control and Prevention, Atlanta, Georgia, United States of America; 4 Malawi-Liverpool-Wellcome Trust Clinical Research Programme, College of Medicine, University of Malawi, Blantyre, Malawi; 5 Center for Global Vaccine Research, Institute of Infection and Global Health, University of Liverpool, University of Liverpool, Liverpool, United Kingdom; 6 International Vaccine Access Center, Department of International Health, Johns Hopkins Bloomberg School of Public Health, Baltimore, Maryland, United States of America; 7 Department of Gastrointestinal Sciences, Christian Medical College, Vellore, Tamil Nadu, India; 8 Instituto Nacional de Ciences Médicas y Nutrición Salvador Zubirán, Mexico City, Mexico; 9 Department of Epidemiology of Microbial Diseases, Yale School of Public Health, New Haven, Connecticut, United States of America; National Institutes of Health, UNITED STATES

## Abstract

Cohort studies, randomized trials, and post-licensure studies have reported reduced natural and vaccine-derived protection against rotavirus gastroenteritis (RVGE) in low- and middle-income countries. While susceptibility of children to rotavirus is known to vary within and between settings, implications for estimation of immune protection are not well understood. We sought to re-estimate naturally-acquired protection against rotavirus infection and RVGE, and to understand how differences in susceptibility among children impacted estimates. We re-analyzed data from studies conducted in Mexico City, Mexico and Vellore, India. Cumulatively, 573 rotavirus-unvaccinated children experienced 1418 rotavirus infections and 371 episodes of RVGE over 17,636 child-months. We developed a model that characterized susceptibility to rotavirus infection and RVGE among children, accounting for aspects of the natural history of rotavirus and differences in transmission rates between settings. We tested whether model-generated susceptibility measurements were associated with demographic and anthropometric factors, and with the severity of RVGE symptoms. We identified greater variation in susceptibility to rotavirus infection and RVGE in Vellore than in Mexico City. In both cohorts, susceptibility to rotavirus infection and RVGE were associated with male sex, lower birth weight, lower maternal education, and having fewer siblings; within Vellore, susceptibility was also associated with lower socioeconomic status. Children who were more susceptible to rotavirus also experienced higher rates of rotavirus-negative diarrhea, and higher risk of moderate-to-severe symptoms when experiencing RVGE. Simulations suggested that discrepant estimates of naturally-acquired immunity against RVGE can be attributed, in part, to between-setting differences in susceptibility of children, but result primarily from the interaction of transmission rates with age-dependent risk for infections to cause RVGE. We found that more children in Vellore than in Mexico City belong to a high-risk group for rotavirus infection and RVGE, and demonstrate that unmeasured individual- and age-dependent susceptibility may influence estimates of naturally-acquired immune protection against RVGE.

## Author summary

Differences in susceptibility can help explain why some individuals, and not others, acquire infection and exhibit symptoms when exposed to infectious disease agents. However, it is difficult to distinguish between differences in susceptibility versus exposure in epidemiological studies. We developed a modeling approach to distinguish transmission intensity and susceptibility in data from cohort studies of rotavirus infection among children in Mexico City, Mexico, and Vellore, India, and evaluated how these factors may have contributed to differences in estimates of naturally-acquired immune protection between the studies. Given the same exposure, more children were at high risk of acquiring rotavirus infection, and of experiencing gastroenteritis when infected, in Vellore than in Mexico City. The probability of belonging to this high-risk stratum was associated with well-known individual factors such as lower socioeconomic status, lower birth weight, and incidence of diarrhea due to other causes. We also found the risk for rotavirus infections to cause symptoms declined with age, independent of acquired immunity. These findings can, in part, account for estimates of lower protective efficacy of acquired immunity against rotavirus gastroenteritis in high-incidence settings, mirroring estimates of reduced effectiveness of live oral rotavirus vaccines in low- and middle-income countries.

## Introduction

Rotavirus is the leading source of gastrointestinal disease burden in children globally, with nearly 10 million severe cases and 193,000 fatalities estimated to occur annually [1]. One decade after their rollout in high-income settings, live oral rotavirus vaccines are currently being introduced to national immunization programs of low- and middle-income countries (LMICs). However, randomized controlled trials and post-licensure studies have reported lower vaccine efficacy and effectiveness against rotavirus gastroenteritis (RVGE) in LMICs compared to higher-income settings [2,3]. Understanding this performance gap is essential to maximizing the impact of rotavirus vaccines where they are needed most.

Recent observational studies have investigated how factors such as oral polio vaccine co-administration [4,5], exposure to breast milk antibodies [6], environmental enteropathy [7], and nutritional status [8,9] influence susceptibility of children to RVGE and performance of oral vaccines. Variation in susceptibility among individuals within and between studies—due to these or other unmeasured risk factors—is well known to influence estimates of vaccine efficacy and effectiveness [10–13]. Differential removal of highly-susceptible individuals to a partially-immune state constitutes a form of frailty bias or effect modification that may persist even in randomized studies [10,14,15]; we use the term bias here in reference to discrepancies between common measures of association, such as hazard ratios and risk ratios, and the per-exposure biological effect of immunity (from vaccination or natural infection) on infection and/or disease endpoints [16,17]. Demonstrations of the impact of variation in susceptibility have arisen in both experimental and theoretical studies [18,19]. Refinements in our ability to characterize such variation both statistically and experimentally [20–27], together with formalizations of per-exposure measures of intervention efficacy in trials [28–30] and observational studies [16,17], have highlighted the potential for heterogeneity in susceptibility to influence epidemiologic measurements.

While the possibility of such frailty bias in rotavirus vaccine studies has been raised [15,31,32], distinguishing its contribution to variation in estimates of vaccine protection against RVGE has been difficult given the concordance of observed patterns with multiple hypotheses [33]. Importantly, the rate of asymptomatic infections and the distribution of risk factors across settings are not easily measured or compared [34], and individual variation in susceptibility may be only partially attributable to known or measured risk factors.

Similarly-designed birth cohort studies undertaken in socioeconomically-distinct LMIC populations of Mexico City, Mexico and Vellore, India provide an opportunity to characterize heterogeneity in susceptibility to rotavirus infection and RVGE, and to assess its influence on estimates of immune protection [35,36]. While the two studies supplied similar estimates of naturally-acquired immune protection against re-infection, differences in estimates of protection against RVGE reflected discrepancies in estimated vaccine efficacy between Latin America and South Asia [37–39]. Whereas no children in Mexico City experienced moderate-to-severe RVGE after two or more previous infections, two previous infections were associated with only 57% protection against moderate-to-severe RVGE among children in Vellore [35,36]. Paired re-analysis of the studies has provided evidence that differences owe, in part, to the influence of a subset of “high-risk” individuals in the Vellore cohort—who experienced high rates of rotavirus infection as well as high risk for RVGE given infection—and age-dependent risk for RVGE given infection [33].

We revisited data from these studies aiming to better understand and compare the distribution of susceptibility among individuals within the two cohorts, and to explore the implications for epidemiologic analyses. We developed a model to estimate susceptibility of children to rotavirus infection and RVGE, accounting for the natural history of rotavirus and differences between settings in transmission intensity. We conducted statistical inference via kernel-based and Markov chain Monte Carlo inference approaches, recovering near-identical parameter estimates under the two strategies. We used our findings to explore the influence of sources of bias underlying conventional measures of protective immunity.

## Results

### Cohort monitoring

**Fig 1:**
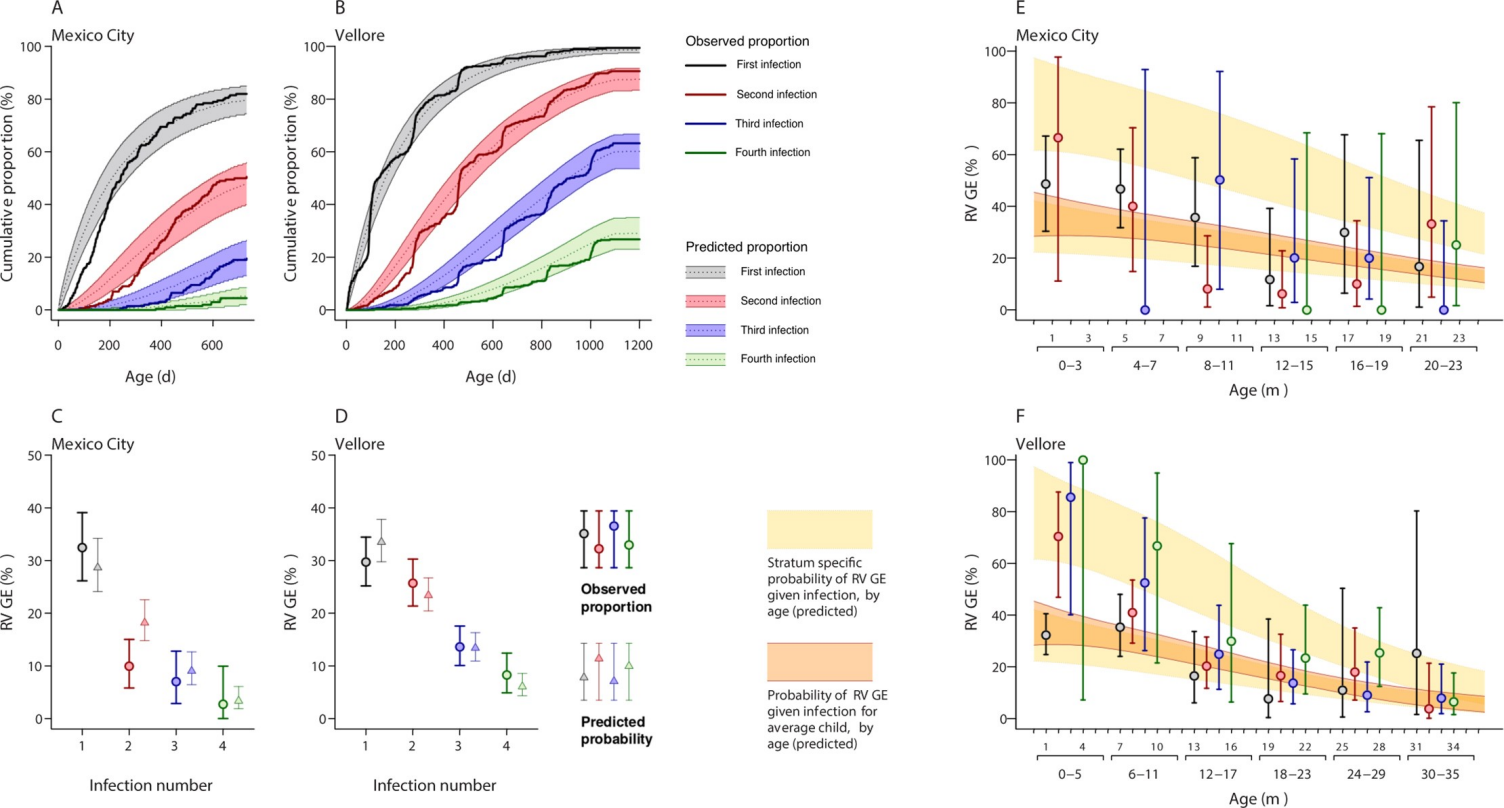
Natural history of rotavirus infection: Observations and model predictions. We plot cumulative incidence of first, second, third, and fourth rotavirus infections by age within the (**A**) Mexico City and (**B**) Vellore cohorts; shaded areas define 95% prediction intervals as fitted by the model, accounting for variation in risk among children and loss to follow-up, with dotted lines indicating median model predictions. (**C** and **D**) Observed proportions (circles, with 95% credible intervals) of infections involving RVGE, and model-predicted probabilities of RVGE given infection (triangles, with 95% prediction intervals), are plotted side by side. (**E** and **F**) We plot age-specific proportions of primary, secondary, third, and later infections involving RVGE (with 95% credible intervals); age strata within which such proportions are calculated correspond to the interval for serological sampling within the two cohorts (four months in Mexico City; six months in Vellore); these age bins are illustrated in the lower half of the x-axis. We superimpose these age-stratified estimates against the continuous decline in risk for infections to cause RVGE estimated by our fitted model (shaded peach); the lower estimate corresponds to the population of a low-risk sub-group (*R*), while the higher estimate corresponds to a higher-risk subgroup (*R*^*C*^) whose risk for RVGE, given infection, is increased by a factor *ρ* (**Table 1**). The intermediate region (shaded orange) denotes risk for the average child within the cohorts. Shaded areas denote 95% prediction intervals around estimates of age-specific risk.

Incidence of rotavirus infection and RVGE among children enrolled in the two cohorts has been described previously [33,35,36,40]. Briefly, the studies enrolled 200 and 373 unvaccinated Mexican and Indian children who were followed from birth to up to 2 years and 3 years of age, respectively, yielding 3699 and 13,937 child-months of follow-up, and characterized the spectrum of asymptomatic to severe clinical manifestations of each rotavirus infection. In total, 315 rotavirus infections were detected in Mexico City and 1103 were detected in Vellore, with 89 (28% of 315 infections) and 282 (26% of 1103 infections) episodes of RVGE occurring in the two settings, respectively. Incidence was higher in Vellore, such that first infections occurred in 56% and 81% of Indian children by ages 6 months and one year, compared to 34% and 67% of Mexican children, respectively (**Fig 1A and 1B**).

The proportion of infections causing RVGE declined with a higher number of previous infections (**Fig 1C and 1D**). However, analyses stratified by age and previous infection revealed this trend could owe to confounding by age, i.e. declining RVGE risk with older age for each of first, second, and later infections (**Fig 1E and 1F**). At matched ages, RVGE was more common, paradoxically, during second and later infections than first infections in Vellore. In contrast, this trend was not apparent in Mexico City.

### Rotavirus natural history

We developed a set of model structures addressing biological hypotheses of rotavirus natural history, based on previous studies of transmission dynamics [41–43] and secondary analysis of the birth-cohort datasets [33]. We estimated the proportion of each cohort belonging to a “high-risk” group, and tested for evidence of variation in susceptibility to infection and/or risk of RVGE given infection among the high-risk group compared to the rest of the cohort (see Materials and Methods). We also tested whether the risk of RVGE given infection varied depending on age at time of infection, and/or the number of previous infections.

We estimated 33% (95% CI: 23% to 41%), 50% (42% to 57%), and 64% (55% to 70%) reductions in the rates at which children re-acquired rotavirus after one, two, or three or more previous infections (**Table 1**), closely recapitulating estimated protection against re-infection in the original studies (**S2 Table**) [35,36].

**Table 1:** Parameter estimates and AIC values from models.

Measure		Definition	Estimate (95% CI)	
			Model 1^2^	Model 2^2^
*α*	Mexico City (M)	Proportion of population belonging to *R* subgroup	0.03 (0.01, 0.38)	0.04 (0.01, 0.46)
	Vellore (V)		0.15 (0.06, 0.39)	0.17 (0.07, 0.49)
*P*(*α*_M_>*α*_V_)		Test for difference between cohorts in prevalence of *R* subgroup (*p* value)	*p =* 0.0594	*p* = 0.0822
*ϕ*		Hazard ratio for infection in *R* subgroup	1.74 (1.27, 2.46)	1.63 (1.15, 2.37)
*ρ*		Relative risk for diarrhea (given infection) in *R* subgroup	2.39 (1.91, 3.14)	2.24 (1.69, 3.12)
*β*_0_		Intercept term, function for RVGE risk given age	-1.05 (-1.31, -0.81)	-1.24 (-1.66, -0.84)
*β*_1_		Linear term, function for RVGE risk given age^1^	-0.16 (-0.52, 0.20)	-0.33 (-0.80, 0.13)
*β*_2_		Quadratic term, function for RVGE risk given age^1^	-0.54 (-1.06, -0.02)	-0.42 (-0.99, 0.14)
*Λ*	Mexico City (M)	Force of infection (baseline infection rate per child per year in the *R*^*c*^ group)	2.27 (1.54, 3.31)	2.11 (1.42, 3.19)
	Vellore (V)		2.55 (1.82, 3.69)	2.37 (1.67, 3.54)
*P*(*Λ*_M_>*Λ*_V_)		Test for difference in force of infection (*p* value)	*p* = 0.0496	*p =* 0.0490
*ψ*_*k*_	*k* = 0	Hazard ratio for infection given previous exposure	1.00 (ref.)	1.00 (ref.)
	*k* = 1		0.67 (0.59, 0.77)	0.67 (0.59, 0.77)
	*k* = 2		0.50 (0.43, 0.58)	0.50 (0.43, 0.58)
	*k* ≥ 3		0.36 (0.30, 0.45)	0.37 (0.30, 0.46)
*ω*_*k*_	*k* = 0	Relative risk for RVGE during infection given previous exposure	1.00 (fixed)	1.00 (ref.)
	*k* = 1		—	1.14 (0.91, 1.44)
	*k* = 2		—	1.15 (0.80, 1.64)
	*k ≥* 3		—	1.30 (0.83, 2.07)
AIC		Akaike information criterion (penalized measure of model fit)	21000.52	21005.38
*e*^–ΔAIC/2^		Relative model likelihood (AIC-penalized)	1	0.088

^1^Linear and quadratic age inputs are centered and scaled to unit variance in the original model.

^2^Model 2 estimates the effect of acquired immune from previous infections on risk of symptoms given reinfection (*ω*_*k*_) controlling for age at infection; Model 1 assumes that the risk of RVGE given infection only depends on age at infection. A third model—wherein such an effect was limited to the *R*^*C*^ risk group—was not identifiable.

Our models also captured declining risk for infections to cause symptomatic RVGE at older ages (**Fig 1B**). The proportion of secondary and subsequent infections causing RVGE in the first year of life in Vellore closely matched expectations among children classified as a “high-risk” subset of the population (detailed below; **Fig 1F**). We compared the fit of models with differing assumptions about acquired immune protection against RVGE given infection (**Table 1**). After accounting for declining risk of RVGE given infection at older ages, we did not identify improvements in fit (based on values of the Akaike Information Criterion [44]) when allowing for acquired immune protection against symptoms during second or later infections (Model 2).

Several salient differences between the two studies were reproduced in model-based predictions. Although we predicted higher-than-observed rates of infection in Mexico City during the first six months of life, predictions accurately reflected between-setting differences in cumulative incidence by the end of the first year (**Fig 1A and 1B**). In addition, fitted parameters recapitulated the observation of significantly lower probabilities of RVGE during second, third, and fourth infections in Mexico City as compared to Vellore (**Fig 1C and 1D**), despite predicting RVGE in a higher-than-observed proportion of second infections in Mexico City.

### Variation in susceptibility among children

Our modeling framework partitioned the cohort populations across distinct risk groups (*R* and *R*^*C*^) with prevalences *α*_M_ and 1–*α*_M_, respectively, in Mexico City, and *α*_V_ and 1–*α*_V_ in Vellore. Because the size of the risk group and group-specific relative risk for infection and/or disease outcomes are inversely related, the relative susceptibility and prevalence of these two risk groups were not simultaneously identifiable. We therefore estimated conditional between-group differences in susceptibility to infection (hazard ratio *ϕ*) and RVGE given infection (relative risk *ρ*) associated with particular values of *α*_M_ and *α*_V_. We reconstructed the full distribution of *ϕ* and *ρ* from the marginal distributions of {*ϕ*,*ρ*}|{*α*_M_,*α*_V_} (see Materials and Methods). Fitting a more complex model (**S1 Text**) which considered an exhaustive set of risk groups—including children with modified susceptibility to infection only or disease only—allowed us to verify the hypothesis of a linkage between children’s susceptibility to infection and disease given infection, which was suggested in previous analyses of the cohort data [33].

**Fig 2:**
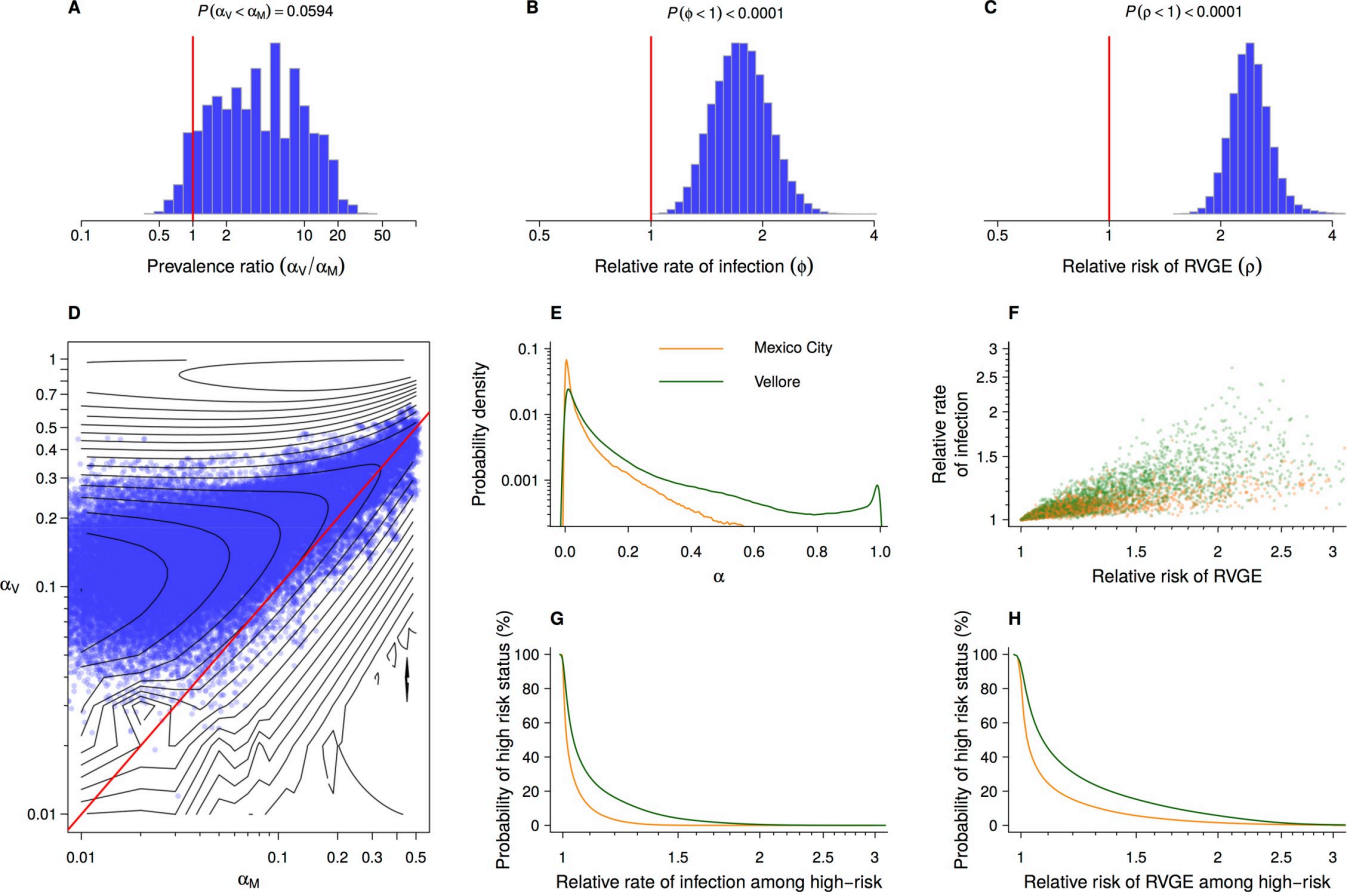
Heterogeneity in susceptibility to rotavirus infection and RVGE within and between cohorts. We plot estimates of (**A**) the relative prevalence of thge “high-risk” subgroup within Vellore (*α*_*V*_) versus Mexico City (*α*_*M*_). For the high-risk subgroup within each cohort, we illustrate (**B**) subgroup-specific hazard ratio of infection (*ϕ*) and (**C**) relative risk of RVGE given infection (*ρ*). (**D**) Sampled points (blue) illustrate the likelihood surface of the joint prevalence of subgroups within the Vellore and Mexico City cohorts; underlying isoclines illustrate the uneven likelihood surface on a natural log scale, while the preponderance of points above the red diagonal suggests that the high-risk subgroup is more prevalent in Vellore than in Mexico City (as indicated in **A**). Near-identical results arise under a model that allows either linked or unlinked susceptibility to infection and disease (**S1 Text**; **S2 Fig**, panel **E**). We next illustrate (**E**) estimates of the absolute sizes of the high-risk subgroups in Vellore (green) and Mexico City (yellow). Sampling from the joint distributions of *α*_*V*_, *α*_*M*_, *ϕ*, and *ρ*, (**F**) we generate simulated cohorts of children from Mexico City (orange points) and Vellore (green) to illustrate their relative susceptibility to infection and to RVGE, given infection. We next illustrate (**G**) the probability of “high-risk” status among children in Mexico City and Vellore associated with increasing values of *ϕ*, the hazard ratio of infection among those who belong to the “high-risk” group. The estimated probability of belonging to the “high-risk” group declines as the group is defined by a more-pronounced risk differential. Similarly, we illustrate (**H**) the declining probability of belonging to the “high-risk” group under increasing values of *ρ*, the relative risk of RVGE given infection among high-risk children.

This modeling approach enabled us to compare the prevalence of children with particular susceptibility levels between cohorts (**Fig 2**). We estimated that 3% (1% to 23%) of children in Mexico City would belong to a high-risk-stratum experiencing a ≥50% higher-than-baseline rate of acquiring rotavirus infection, compared to 13% (6% to 29%) of children in Vellore (**Fig 2G**). A subgroup with over double the baseline rate of infection would include 2% (1% to 8%) of children in Mexico City and 10% (5% to 18%) of children in Vellore, while only 1% (0% to 2%) of children in Mexico City and 6% (5% to 9%) of children in Vellore would belong to a subgroup experiencing rates of infection ≥3-fold higher than the baseline rate. Greater susceptibility to infection was associated with higher risk of experiencing RVGE given infection, regardless of the prevalence of the high-risk group (**Fig 2F**); fitting both *ϕ* and *ρ*, we identified ≥99.99% probability for excess risk of disease given infection within the sub-cohorts defined to have higher rates of acquiring infection (**S1 Table**). Joint distributions of *ϕ* and *ρ* with the size of the risk groups were indistinguishable under the original model specification and a more complex model that allowed either linked or unlinked susceptibility to infection and disease (**S1 Text, S2 Fig**).

### Determinants of susceptibility among individual children

Our modeling approach provided a statistical basis for calculating the probability that each child belonged to the “high-risk” subgroup (see Materials and Methods). To examine the validity of these estimates, we next assessed whether a child’s estimated risk of belonging to the “high-risk” subgroup was related to host factors and exposures measured in the original studies that have previously been reported to predict risk for rotavirus infection and RVGE (**Tables 2 and 3**).

**Table 2:** Characteristics of the two cohorts.

Observation		Mexico City	Vellore
Birth weight	(kg)	3.2 (median; IQR: 2.9, 3.4)	3.0 (median; IQR: 2.7, 3.2)
Weight at 12 mo.	(kg)	10.0 (median; IQR: 9.1, 10.6)	7.9 (median; IQR: 7.3, 8.6)
Sex	Female	94 (47.0%)	186 (49.9%)
	Male	106 (53.0%)	187 (50.1%)
Duration of breastfeeding^1^			
	<3 months	59 (38.1%)	15 (4.1%)
	3–5 months	27 (17.4%)	27 (7.3%)
	6–11 months	31 (20.0%)	56 (15.1%)
	12+ months	38 (24.5%)	272 (73.5%)
Number of siblings			
	0	66 (33.8%)	118 (31.6%)
	1	56 (28.7%)	123 (33.0%)
	2	38 (19.5%)	90 (24.1%)
	3+	35 (17.9%)	42 (11.3%)
Socioeconomic status			
	Lowest	24 (12.3%)	– –
	Intermediate	81 (41.5%)	– –
	Highest	90 (46.2%)	– –
	Not bidi-working household	– –	200 (53.6%)
	Bidi-working household	– –	173 (46.4%)
Maternal education^2^			
	0–4 years	57 (29.2%)	106 (28.4%)
	5–7 years	62 (31.8%)	109 (29.2%)
	8–9 years	46 (23.6%)	90 (24.1%)
	≥10 years	30 (15.4%)	68 (18.2%)
RVGE and symptom severity	Children experiencing any RVGE episode(s)	77 (38.5%)	178 (47.7%)
	Children experiencing RVGE with Vesikari score ≥11	14 (7.0%)	58 (15.5%)
Episodes of rotavirus-negative diarrhea^3^		4 (median; IQR: 2, 7)	4 (median; IQR: 2, 6)
Episodes of respiratory infection		– –	20 (median; IQR: 14, 26)

All percentages are calculated among children from whom data were available for the given variable. IQR: interquartile range.

^1^Calculated among children retained for over 1 year in the study.

^**2**^Breakpoints for the years of maternal education were defined according to the following standard for Vellore: 0–4, no formal education or primary school incomplete; 5–7, primary school complete; 8–9, middle school complete; 10 or more, high school complete.

^3^Duration of follow-up differs by setting (up to 2 years in Mexico City, versus up to 3 years in Vellore).

**Table 3:** Factors associated with belonging to the “high-risk” group.

Risk factor		Relative Risk (95% CrI)		
		Mexico City	Vellore	Pooled analysis
Birth weight	per log kg	0.68 (0.42, 0.94)	0.84 (0.58, 1.13)	0.47 (0.20, 0.98)
Weight at 12 mo.	per log kg	0.88 (0.74, 1.01)	1.00 (0.85, 1.16)	0.68 (0.23, 1.66)
Sex	Female	ref.	ref.	ref.
	Male	1.05 (0.79, 1.42)	1.38 (1.08, 2.03)	1.26 (1.04, 1.67)
Duration of breastfeeding^1^				
	<3 months	ref.	ref.	ref.
	3–5 months	0.69 (0.41, 1.07)	1.12 (0.434, 3.04)	0.79 (0.50, 1.20)
	6–11 months	0.91 (0.54, 1.44)	1.16 (0.480, 2.86)	0.91 (0.59, 1.37)
	12+ months	0.70 (0.42, 1.05)	1.11 (0.485, 2.51)	0.83 (0.54, 1.17)
Number of siblings				
	0	ref.		ref.
	1	0.88 (0.59, 1.27)	0.72 (0.455, 0.98)	0.76 (0.52, 0.97)
	2	0.69 (0.42, 0.98)	0.79 (0.509, 1.16)	0.75 (0.53, 1.00)
	3+	0.49 (0.28, 0.74)	0.74 (0.413, 1.12)	0.61 (0.40, 0.83)
Socioeconomic status				
	Lowest	ref.	– –	– –
	Intermediate	1.20 (0.78, 1.92)	– –	– –
	Highest	1.17 (0.77, 1.78)	– –	– –
	Not bidi-working household	– –	ref.	– –
	Bidi-working household	– –	1.45 (1.095, 2.22)	– –
Maternal education				
	0–4 years (primary school incomplete)	ref.	ref.	ref.
	5–7 years (primary school complete)	1.21 (0.86, 1.80)	0.89 (0.62, 1.28)	0.99 (0.76, 1.32)
	8–9 years (middle school complete)	1.14 (0.77, 1.76)	0.90 (0.61, 1.35)	0.98 (0.73, 1.34)
	≥10 years (high school complete)	1.13 (0.75, 1.80)	0.62 (0.36, 0.90)	0.75 (0.50, 1.00)
Severity of RVGE episodes	Any episode with Vesikari score ≥11 (among children experiencing RVGE)	1.17 (0.82, 1.97)	1.52 (1.13, 2.55)	1.44 (1.13, 2.22)
Incidence of rotavirus-negative diarrhea	per log episodes/year	1.07 (1.02, 1.16)	1.38 (1.17, 1.72)	1.15 (1.07, 1.28)
Incidence of respiratory infections	per log episodes/year	– –	1.13 (1.10, 1.19)	– –

^1^Only children retained in studies for over 1 year are included in analyses.

Male children were 26% (4% to 67%) more likely to be among the “high-risk” subgroup than female children (**Table 3**). Birth weight was also a predictor of being in the “high-risk” subgroup, with each log-kilogram decrease in birth weight conferring 2.05 (1.02 to 5.03)-fold higher probability of belonging to the “high-risk” subgroup. However, we did not detect a significant association between susceptibility and weight at 12 months. In each cohort as well as in the pooled analysis, children without siblings were more likely to belong to the “high-risk” subgroup than children with siblings. In comparison to children whose mothers had completed <5 years of education, children whose mothers had completed ≥10 years of education were 25% (0% to 50%) less likely to belong to the “high-risk” subgroup.

We also identified several factors predicting within-cohort variation in susceptibility that were consistent with findings in primary analyses of the studies [35,36]. In Vellore, children whose household members were involved in producing bidis (indigenous cigarettes)—an indicator of lower household socioeconomic status—were 45% (10% to 122%) more likely than other children to belong to the “high-risk” subgroup. In Mexico City, children with a shorter duration of breastfeeding were more likely to belong to the “high-risk” subgroup, although this association did not reach conventional thresholds of statistical significance in our analysis.

Among children experiencing RVGE in the cohorts, those who experienced moderate-to-severe RVGE symptoms (defined by a Vesikari score ≥11) on at least one episode were 44% (13% to 122%) more likely to belong to the “high-risk” subgroup, suggesting model-based measures of susceptibility to rotavirus infection and (any) RVGE given infection also predicted the severity of rotavirus disease. In addition, children who experienced higher rates of diarrheal episodes caused by pathogens other than rotavirus were more likely to belong to the “high-risk” subgroup within each cohort. In Vellore, we also found a positive association between the incidence of acute respiratory infections and the likelihood that a child belonged to the “high-risk” subgroup; this information was not available for the Mexico City cohort.

### Sources of variation in estimates of naturally-acquired immune protection

**Fig 3:**
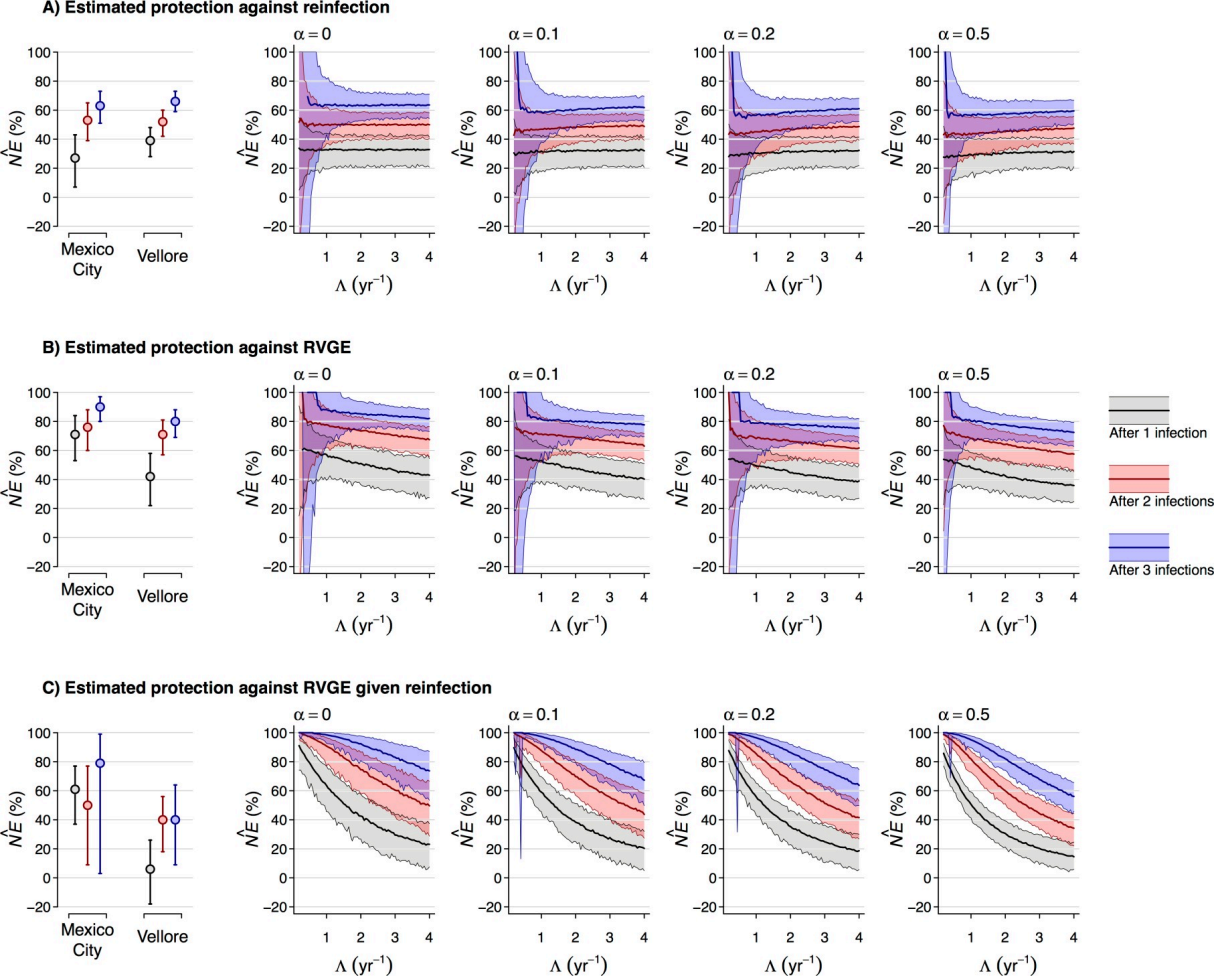
Sources of bias in estimates of naturally-acquired immune protection. We compare “naïve” estimates of naturally-acquired immunity ($\hat{NE}$) against re-infection (**A**), against RVGE incidence (**B**), and against RVGE given re-infection (**C**) following one (grey), two (red), or three (blue) previous infections in simulated cohorts. We parameterize the natural history model with estimates of natural protection against re-infection (*ψ*), age-specific risk of RVGE given infection (*β*), and excess infection and RVGE risk within the “high-risk” subgroup (*ϕ*, *ρ*) from Model 1 (**Table 1**, **Fig 2**). We vary the force of infection (*Λ*, measured as infections per susceptible person-year-at-risk) and the proportion of children assumed to belong to the “high-risk” subgroup (*α*). Consistent with primary analyses of the cohort data, simulated (“naïve”) analyses do not account for age-dependent RVGE risk. The plotted values can be interpreted as predictions of estimated natural immunity against rotavirus infection, RVGE, and RVGE given infection, across sites with differing force of infection (x-axes) or prevalence of high-risk individuals (panels left to right). We plot estimates from the original cohort datasets within the left panels of each row (circles, with 95% confidence intervals), revealing that the higher force of infection and prevalence of “high-risk” individuals in Vellore can explain some, although not all, of the difference in estimates of immune protection between the Vellore and Mexico City cohorts.

We next conducted simulation studies (**Fig 3**) to assess how variation in transmission intensity and in the susceptibility of children could influence estimates of naturally-acquired immune protection against rotavirus infection, RVGE, and RVGE given infection [45]—as measured by the hazard ratios of infection and RVGE, and relative risk of RVGE given infection—following one, two, or three previous infections, compared to zero previous infections. We compared estimates from *in silico* cohorts with differing prevalence of “high-risk” children (*α*) exposed to varying forces of infection (*Λ*). We accounted for susceptibility differences between risk groups by sampling from the joint, unconditional distribution of {*ϕ*,*ρ*}, thereby isolating the effect of differences in risk-group prevalence.

We did not identify a large of impact of susceptibility differences on estimates of protection against reinfection (i.e. estimates of $\hat{NE}$ were similar across different levels of *Λ* and *α*), which may help to explain why these estimates were nearly equal in the original studies [35,36]. However, we found that estimates of protection against RVGE—which were lower in primary analyses of the Vellore cohort—were expected to decline in settings with higher transmission intensity, reflecting acquisition of infection at younger, higher-risk ages. The impacts of heterogeneity in susceptibility were outweighed by the impacts of unaccounted-for age-dependent symptom risk. For a population exposed to transmission intensity on the order of one rotavirus infection per susceptible child-year at risk, increasing the prevalence of the “high-risk” subgroup (*α*) from 0% 50% reduced the estimate of protection against RVGE conferred by one previous infection by 9% (–12% to 27%), in absolute terms. Increasing transmission intensity to the equivalent of four infections per susceptible child-year at risk led to a reduction of 14% (–8% to 34%) at *α* = 0 and, similarly, of 12% (–6% to 27%) at *α* = 0.5, in absolute terms.

## Discussion

Evidence of naturally-acquired immunity against rotavirus from birth-cohort studies provided an impetus toward the development of live oral rotavirus vaccines, which are now among the most effective strategies for the prevention of severe illness and deaths due to RVGE globally [46]. However, challenges have persisted in understanding and addressing the lower protective efficacy of rotavirus vaccines in high-burden LMIC settings, which mirrors protection derived from naturally-acquired immunity [47,48]. Our analysis suggests that discrepant estimates of protection may in part reflect epidemiological bias, attributable to differences between settings in transmission intensity and differential susceptibility of children to rotavirus infection and RVGE, individually and by age. Lower estimates of protection in settings with high rotavirus burden thus reflect factors other than weaker immunity among children in LMICs.

Accounting for aspects of the natural history of rotavirus enabled us to directly compare the susceptibility of children enrolled in birth cohort studies undertaken in socioeconomically-distinct settings. Although we estimated only modestly higher susceptibility for the average child in Vellore as compared to Mexico City, individual variation in susceptibility was considerably greater within the Vellore cohort. We estimated that a higher proportion of children in Vellore, as compared to Mexico City, showed elevated rates of rotavirus infection as well as excess risk for RVGE given infection. This finding can account for several unexpected features of the epidemiology of rotavirus in Vellore. The increasing probability of RVGE in association with first, second, and later infections occurring at matched ages that we identified, particularly in Vellore (**Fig 1F**), reflects high risk for RVGE given infection among individuals susceptible to frequent rotavirus infection. In other words, children who experienced two or more infections before 6 months of age, or three or more infections before 12 months of age, are more likely to belong to a subgroup with pronounced susceptibility to rotavirus infection and disease, given infection. Indeed, our analysis identified that susceptibility to rotavirus infection was positively associated with susceptibility to RVGE given infection among individual children (**S1 Table**, **S2 Fig**). While the proportion of children belonging to a “high-risk” subgroup constituted a source of epidemiologic bias in simulation studies, and was expected to lead to estimates of weaker protection against RVGE in settings with higher transmission intensity such as Vellore, the degree of bias imposed was not large.

Several other studies have recently addressed transmission-dynamic factors that may contribute to the apparent underperformance of rotavirus vaccination in high-transmission settings [32]. Using data from the PROVIDE trial of monovalent rotavirus vaccine in Bangladesh, Rogawski and colleagues demonstrated that acquisition of naturally-acquired immunity may contribute to lower estimates of vaccine efficacy due to earlier and more frequent infection within the control arm; impacts on estimates of protection are most notable in high-transmission settings, and among children in their second year of life [15]. Here we were able to account for the contribution of all previous infections to naturally-acquired immunity, including subclinical infections, and to account for age-specific RVGE risk. Selection bias resulting from variation in individual susceptibility can result in further downward bias [31], underscoring the need for per-exposure estimates of immune effectiveness such as we have sought in this analysis. Directly comparing susceptibility between populations or settings is difficult because determinants of susceptibility are often unknown or unmeasured, and may be imperfectly characterized by measurable epidemiological risk factors. The contributions of susceptibility and transmission intensity to disease incidence rates are not easily disentangled. Our analysis employed a novel approach to characterize susceptibility of children in two cohorts based on a model that included known aspects of rotavirus natural history, facilitated by access to similar measurements from settings with distinct risk profiles and force of infection.

Our estimates of susceptibility appear externally valid based on their association with previously-reported risk factors for RVGE [49–52]: male children, children with lower birth weight, and children whose mothers had lower educational attainment were more likely to belong to a higher-risk subset of the population in our analysis. In Vellore, children whose households were involved in bidi work—a marker for lower socioeconomic status—were also at higher risk [36], while in Mexico City, we observed a trend toward lower risk associated with longer breastfeeding, consistent with previous studies [53,54]. In addition, we observed higher incidence of diarrhea caused by pathogens other than rotavirus among children who were found to have greater susceptibility to rotavirus. This observation may signify the presence of environmental enteric dysfunction within the cohorts, or other sources of variation in immune status or pathogen exposure. In Vellore, children who we estimated were more susceptible to rotavirus also experienced higher incidence of respiratory infections, as reported previously [36]. While the associations we identify (in particular with time- or age-specific risk factors) do not measure causal effects in either direction, our inferences pertaining to within-cohort susceptibility are supported by the fact that children classified by the model as having “high risk” exhibit risk factors widely believed to be associated with rotavirus infection and RVGE.

These and other host factors associated with susceptibility to rotavirus infection and RVGE have also been reported to predict weaker immune responses to live enteric vaccines such as those against rotavirus. While our model does not address variation in the strength of immune responses among individuals or across settings, 58% of Indian children versus 90% of Mexican children seroconverted after Rotarix immunization in previous studies [47,55]. Nonetheless, near-equal naturally-acquired protection against re-infection was noted among children in the birth cohort studies in Vellore and Mexico City. Our findings demonstrate that some degree of the reported variation in protection against RVGE can be attributed to epidemiological biases resulting from differential transmission intensity and differential susceptibility of children, although we found age-dependent diarrhea risk was a more important contributor to variation in estimates.

The finding that older age diminishes risk for children to experience RVGE given rotavirus infection has been suggested in previous analyses of the cohort datasets [33]. Our simulation study demonstrates that such age-related symptom risk enhances protection against RVGE in low-transmission settings. Deferring infections to later ages significantly reduces the risk for children to experience symptoms upon reinfection. While the mechanisms underlying age-dependent diarrhea risk are not precisely known, the observation has been reported in mouse, rat, rabbit, and gnotobiotic piglet models of rotavirus infection [56–59]. Age-dependent TLR3 expression and host responses to rotavirus enterotoxins contribute to this observation in mice [60,61]. Other aspects of immune maturation, intestinal development, and the establishment of gut microbial communities may further drive associations between age and diarrhea risk in both humans and animals [62]. Furthermore, the greater dehydrating effect of diarrhea in younger children with smaller body volumes may contribute to severity—and thus the reporting and diagnosis—of RVGE in early-life infections [63].

There are several limitations to our analysis. Whereas we assume exponentially-distributed infection times (consistent with a constant hazard of infection), this provides an imperfect fit to the timing of early-life infections, particularly in the Mexico City cohort. The departure between predictions and observations may reflect the protective effect of maternal antibodies, as reported previously [35,64], or the influence of age-specific social mixing patterns on transmission [65]. Thus, our model tended to overestimate the probability of RVGE associated with second rotavirus infections in Mexico City, although this discrepancy was not sustained for third and fourth infections. Our analyses also do not distinguish between homotypic and heterotypic protection because we lack genotype data for serologically-detected infections, which constitute the majority of infections in both cohorts. Although moderate-to-severe RVGE episodes are the primary endpoint of most studies evaluating vaccine efficacy and effectiveness, our analysis addressed RVGE episodes of any severity. Only 7% of children in Mexico City experienced RVGE episodes with Vesikari score ≥11, limiting the statistical power for analyses of moderate-to-severe RVGE. Nonetheless, previous analyses of the studies identified similar risk factors for mild and moderate-to-severe RVGE [33]; moreover, we find that children identified by our method to face higher risk for rotavirus infection and RVGE likewise experienced higher risk for moderate-to-severe manifestations of RVGE episodes. Thus, our findings may inform the interpretation of studies with moderate-to-severe RVGE endpoints.

Our ability to account for variation in susceptibility to infection as well as disease, and indeed to identify a linkage between these traits, is a unique advantage afforded by data describing both clinically-apparent and subclinical infections. While methods exist to account for frailty in time-to-event data [10,66], as may be present in studies with only one class of endpoints (such as serological studies of infection or clinical trials with disease endpoints), susceptibility to disease given infection is also of interest. Importantly, our findings suggest that age, rather than naturally-acquired immunity, determines risk for rotavirus infections to present symptomatically, together with individual-level susceptibility factors. Adaptations of our model to the natural history of other pathogens may facilitate similar studies in other disease-specific contexts. Whereas other models have used continuous distributions to characterize individual susceptibility, this approach has generally relied on the ability to measure or even manipulate exposure intensity at the individual level, for instance by measuring infectious contacts or through controlled-dose challenge experiments [20–30]. As our data presented the opportunity to compare exposure intensity between cohorts but not between individuals, we considered a simpler case of dichotomous risk groups within cohorts, and determined how the sizes of cohort-specific risk strata (*α*) were jointly distributed with the degree of risk elevation (*ϕ*,*ρ*). *A priori* knowledge of risk strata, for instance based on previous estimates of covariate effect sizes, presents an alternative strategy to infer distributions of individual-level susceptibility [67].

Birth-cohort studies have been instrumental to our understanding of the natural history of rotavirus. Uncertainties surrounding differences in the epidemiology of rotavirus in socioeconomically-distinct populations underscore the need for a theoretical basis for comparing outcomes of individual studies. Our approach permitted assessment of how age, acquired immunity, and variation in individual susceptibility independently contributed to infection and disease risk in distinct birth cohorts, helping to resolve discrepancies in estimated protection that arose in primary analyses of the datasets. The modeling framework we introduce here may thus have applicability to studies of other partially-immunizing pathogens.

## Materials and methods

### Birth cohort data

The two birth cohort studies followed similar protocols that have been described previously [35,36]. Children were enrolled at birth and followed to 24 and 36 months of age in Mexico City and Vellore, respectively. The studies aimed to detect all rotavirus infections, both symptomatic and asymptomatic. Rotavirus infections were detected by three approaches: (1) sera were drawn every 4 and 6 months in Mexico City and Vellore, respectively, and tested for IgA or IgG titer increases; (2) asymptomatic stool samples were collected weekly in Mexico City and every two weeks in Vellore and tested for rotavirus; and (3) diarrheal stools were collected by field workers every time mothers alerted the study teams of any change in a child’s stool pattern (**S2 Table**). Virus detection was performed by ELISA in Mexico City and by ELISA or real-time PCR in Vellore. In Mexico City, 200 children were recruited and retained for 77% of the scheduled follow-up period, while our analysis of the Vellore dataset included the 373 children (83% of 452 enrolled) who completed follow-up. Data were available for 96% (1037/1080) and 99% (2565/2598) of scheduled serum tests; 97% (15,503/16,029) and 93% (26,902/28,906) of scheduled asymptomatic stool tests; and 85% (963/1133) and 99% (1829/1856) of reported diarrheal episodes in Mexico City and Vellore, respectively.

### Modeling rotavirus natural history

#### Summary

We developed a probabilistic model describing rotavirus natural history that allowed us to test how susceptibility to rotavirus infection and RVGE vary innately among individuals and according to age and previous infection. Seeking to account for differences in rotavirus epidemiology in the two settings under a unified model, we assumed parameters reflecting the strength of immune protection and the effect of age on the probability of symptoms given infection were equal in both settings, whereas we permitted the force of infection and the proportion of children belonging to distinct risk strata to vary between settings. We compared the improvement in fit afforded by modifications reflecting distinct hypotheses about the acquisition of protection against symptoms given infection—a source of uncertainty in rotavirus epidemiology [33]. Individual-level susceptibility of children to infection and RVGE was measured from the probability for children to belong to the high-risk stratum, which we also tested for associations with epidemiological risk factors. Last, we simulated from the fitted model to explore how differences in transmission intensity and the prevalence of high-risk individuals influence estimates of protection.

#### Model

We developed a model for time-to-event data that included asymptomatic and symptomatic rotavirus infection endpoints. Event time distributions were modeled from estimates of the force of infection and from rotavirus natural history parameters modifying the rate at which individuals acquired and re-acquired rotavirus infection, as well as the probability of experiencing RVGE given infection. Observations from previous analyses of the cohort studies suggested individuals differ in their susceptibility: in Vellore, children who experienced diarrhea on second and later rotavirus infections were more likely to have experienced diarrhea on earlier-life infections [33]. We therefore accounted for potential variation in risk among children by defining proportions *α*_V_ and *α*_M_ of children in Vellore and Mexico City, respectively, belonging to a distinct risk group *R*, and tested for group-wise differences in susceptibility to rotavirus infection and RVGE in the *R* stratum relative to the remaining population (*R*^*c*^, with prevalence 1–*α*_*s*_ in setting *s*). We defined the *R* stratum as whichever sub-group occupied ≤50% of the Mexico City cohort, and thus, 0≤*α*_*M*_≤0.5 and 0≤*α*_*V*_≤1; the designation of the *R* stratum as lower- or higher-risk depended upon estimates of the relative susceptibility of children to infection and disease (*ϕ* and *ρ*, respectively; **Table 1**), the inverse of which would apply for the remaining children. In a supplemental analysis (**S1 Text**), we considered the possibility of additional risk strata, which included all pairwise combinations of modified susceptibility to infection and/or modified susceptibility to RVGE given infection.

Whereas primary analyses of the cohort datasets identified decreasing RVGE incidence rates following previous rotavirus infection, re-analyses suggested this declining risk resulted from naturally-acquired protection against rotavirus infection and older age at time of reinfection [33]. We used the Akaike Information Criterion (AIC) [44] to compare the fit of alternative, nested models premised on differing assumptions about the influence of age and naturally-acquired immunity on risk of experiencing diarrhea symptoms during rotavirus infection. Model 1 assumed the risk of RVGE given infection did not vary based on the number of previous infections. Model 2 considered both age and previous infection as determinants of risk for RVGE given infection. We also attempted to fit a third model assuming that only children in the *R*^*C*^ (effectively, lower-risk) group acquired protection against symptoms given infection, but found that parameters were not identifiable; thus, only Models 1 and 2 were considered.

We modeled infection at the rate $\lambda_{R^{c},s,k}=\Lambda_{s}\psi_{k}I_{k}$ among children in the majority (*R*^*c*^) risk group, where *Λ*_*s*_ was the force of infection in Mexico City or Vellore, *I*_*k*_ was an indicator that an individual had experienced *k* previous rotavirus infections, and *ψ*_*k*_ was the hazard ratio for reinfection resulting from naturally-acquired immune protection following *k* previous infections. Among children in the minority (*R*) risk group, we modeled $\lambda_{R,s,k}=\phi \lambda_{R^{c},s,k}$, where the hazard ratio *ϕ* conveyed the effect of differential susceptibility to infection.

The probability of RVGE given rotavirus infection, *π*, was modeled as a function of age and risk group under Model 1. For children of age *x* in the majority risk group,

$$\pi_{R^{c}}^{\left(1\right)}(x)=\exp\left(\beta_{0}+\beta_{1}x+\beta_{2}x^{2}\right),$$ (1)

where the parameters *β*_0–2_ related RVGE risk to the child’s age. Linear, exponential, and higher-order polynomial functions relating risk to age were explored, but found not to improve model fit via AIC. For the remaining children,

$$\pi_{R}^{\left(1\right)}(x)=\rho exp(\beta_{0}+\beta_{1}x+\beta_{2}x^{2}),$$ (2)

where the relative risk *ρ* accounted for innate differences in susceptibility to RVGE given infection.

Under Models 2 and 3, we further accounted for variation in symptom risk according to the number of previous infections a child in the *R*^*c*^ stratum has experienced, such that for Model 2,

$$\begin{array}{l} \pi_{R^{C}k}^{\left(2\right)}\left(x\right)=\exp\left(\beta_{0}+\beta_{1}x+\beta_{2}x^{2}\right)\omega_{k}I_{k},\\ \pi_{Rk}^{\left(2\right)}\left(x\right)=\rho \exp\left(\beta_{0}+\beta_{1}x+\beta_{2}x^{2}\right)\omega_{k}I_{k}, \end{array}$$ (3)

where *ω*_*k*_ indicated the fold change in risk of RVGE given infection after having experienced *k* previous infections. For Model 3,

$$\pi_{R^{c},k}^{\left(3\right)}(x)=\exp(\beta_{0}+\beta_{1}x+\beta_{2}x^{2})\omega_{k}I_{k},$$ (4A)

whereas

$$\pi_{R,k}^{\left(3\right)}(x)=\rho exp(\beta_{0}+\beta_{1}x+\beta_{2}x^{2})$$ (4B)

for all *k*.

#### Likelihood

We estimated model parameters in a likelihood-based framework. Observations included all infections or instances of censoring (indexed by *j*) for each child (indexed by *i*). For the *j*th observation of the *i*th child, we denoted time since the last observation or birth as *Δt*_*ij*_, indicating whether the child was currently infected with rotavirus with the variable *Z*_*ij*_ (= 1 if infected and 0 if censoring occurred) and experiencing RVGE with the variable *D*_*ij*_ (= 1 if RVGE occurred upon infection and 0 otherwise). The likelihood contribution of observation *j* from child *i*, conditioned on the child belonging to either the *R* or *R*^*c*^ subgroup (*) and residing in setting *s*, was

$$L(i,j|*,s)=f(\Delta t_{ij}|\lambda_{*sk_{j}})\left[\pi_{*k_{j}}(x_{ij})D_{ij}+(1-\pi_{*k_{j}}(x_{ij}))(1-D_{ij})\right]Z_{ij}+[1-F(\Delta t_{ij}|\lambda_{*{sk}_{j}})](1-Z_{ij}).$$ (5)

The term $f(\Delta t_{ij}|\lambda_{*sk_{j}})$ represents the probability that the time to infection for individual *i* was *Δt*_*ij*_, while $1-F(\Delta t_{ij}|\lambda_{*{sk}_{j}})$ was the probability for child *i* to have escaped infection from the time of the previous observation to the last follow-up visit, if censoring occurred at the *j*th observation. We assumed an exponentially-distributed time to infection for inference, consistent with an assumption of independent time-to-event observations given the child’s exposure, infection history, and susceptibility status.

We obtained the likelihood contribution of each child, *H*_*s*_(*i*), via the total probability

$$H_{s}(i)=\alpha_{s}\prod_{j}L(i,j|R,s)+(1-\alpha_{s})\prod_{j}L(i,j|R^{c},s).$$ (6)

Defining **Y**_*i*_ as the set of observations from each child *i*, the probability for any particular child to be in the *R* risk group was

$$P(R|Y_{i},s)=\frac{P(R\cap Y_{i}|s)}{P(Y_{i}|s)}=\frac{\alpha_{s}P(Y_{i}|R,s)}{\alpha_{s}P(Y_{i}|R,s)+(1-\alpha_{s})P(Y_{i}|R^{c},s)}=\frac{\alpha_{s}\prod_{j}L(i,j|R,s)}{\alpha_{s}\prod_{j}L(i,j|R,s)+(1-\alpha_{s})\prod_{j}L(i,j|R^{c},s)}.$$ (7)

Our statistical framework assumed that children were sampled at random from the population and that observations **Y**_*i*_ were randomly-assigned given the setting, age, risk group, and infection history of an individual child *i*, such that the sample proportion of children in the *R* group was expected to converge to the population proportion.

We thus defined the overall model likelihood in two components. The first, $G_{1}^{s}=\prod_{i}H_{s}(i)$, conveyed the direct contributions of exponentially-distributed time-to-event observations **Y**_*i*_ as described above. The second component, $G_{2}^{s}$, reflected concordance between the population proportion, *α*_*s*_, and the sample proportion, $\frac{1}{N}\sum_{i}P(R|Y_{i},s)$ of children in the *R* risk group. We calculated $G_{2}^{s}$ by evaluating the probability density of *α*_*s*_ under the assumption of random assignment:

$$\alpha_{s}∼\mathrm{Beta}\{\sum_{i}P(R|Y_{i},s),\sum_{i}\left[1-P(R|Y_{i},s)\right]\}.$$ (8)

Altogether, the model likelihood was the product of these terms across the two settings, $G_{1}^{M}G_{1}^{V}G_{2}^{M}G_{2}^{V}$.

#### Estimation

Estimated between-group differences in susceptibility to infection and disease (*ϕ* and *ρ*, respectively) were dependent upon the size of the underlying proportions *α*_M_ and *α*_V_. For efficiency, we used a kernel-based inference approach whereby we reconstructed the global distribution of parameters from their conditional distributions *θ* = {*ϕ*, *ρ*, *ψ*_1−3_, *ω*_1−3_, *β*_0−2_, *Λ*_M_, *Λ*_V_} given fixed values of {*α*_M_, *α*_V_}, defined “locally” for *α*_M_ = 0, 0.01, …, 0.5, and *α*_V_ = 0, 0.01, …, 1, respectively. For each set {*α*_M_, *α*_V_}, we determined the conditional maximum likelihood parameter estimates θ*|*α*_M_,*α*_V_ via the Nelder-Mead algorithm, using the optim() function in R:

$$\theta^{*}|\alpha_{M},\alpha_{V}={\mathrm{argmin}}_{\theta |\alpha_{M},\alpha_{V}}\left(-\ln(G_{1}^{M}G_{1}^{V}G_{2}^{M}G_{2}^{V})\right).$$ (9)

We assumed local parameter distributions at each set {*α*_M_, *α*_V_} were multivariate-normal, with means defined by the conditional maximum likelihood estimates, and the asymptotic covariance matrix defined from the inverse of the negative Hessian, as evaluated by minimizing the negative log likelihood. We pooled draws from these conditional (local) multivariate normal kernels over all {*α*_M_, *α*_V_}, weighting by their respective likelihoods, to recover the unconditional (global) parameter distributions.

Independent estimation of *ϕ* and *ρ*, conditional on {*α*_M_, *α*_V_}, further enabled us to test the hypothesis of a linkage between susceptibility to infection and disease, as suggested by the finding of higher diarrhea risk among children experiencing high rates of re-infection (**Fig 1E and 1F**) and previously-reported associations [33]. Estimation of these parameters allows us discriminate among hypotheses about susceptibility to infection and disease within the cohorts:

1. That there is no variation in susceptibility to infection or disease (*ϕ =* 1 and *ρ =* 1);
2. That excess/diminished susceptibility to infection has no linkage to disease risk (*ϕ≠*1 and *ρ =* 1), or that excess/diminished susceptibility to disease has no linkage to infection risk (*ρ≠*1 and *ϕ =* 1);
3. That susceptibility to infection and disease are inversely correlated (*ρ>*1 and *ϕ<*1, or *ρ<*1 and *ϕ>*1); or
4. That susceptibility to infection and disease are, in fact, correlated (*ρ>*1 and *ϕ>*1, or *ρ<*1 and *ϕ<*1).

We measured the probability of a linkage between these traits, as suggested under Hypothesis 4, as

Pr(ρ>1|ϕ>1)Pr(ϕ>1)+Pr(ρ<1|ϕ<1)Pr(ϕ<1). (10)

#### Supplemental analysis

We verified parameter estimates from this kernel-based approach in an additional analysis, wherein we sampled from the distribution of parameter estimates via Markov chain Monte Carlo using a Metropolis-Hastings updating procedure. Under this approach, we were able to explore an exhaustive set of risk strata wherein susceptibility to infection and to RVGE given infection could be linked or unlinked. This approach yielded near-identical parameter estimates and inferences in comparison to the original kernel-based approach, as we detail in the supporting files (**S1 Text**, **S1 Table**, **S1 Fig**, **S2 Fig** and **S3 Fig**); the size of the risk groups not considered in the original analysis converged to zero. We identified strong evidence against the null hypothesis of unlinked susceptibility to infection and disease, with posterior probability for the null hypothesis less than 6.7×10^−6^.

### Determinants of individual risk

To better understand variation in susceptibility among children within each cohort, we evaluated associations between individual-level factors (**Table 2**) and the probability for each child to belong to the high-risk (*R*) subgroup. For each of 10,000 draws of *θ*, we measured the probability of belonging to the high-risk group, equal to *P*(R|**Y**_*i*_,*s*) for *ρ*>1 or 1−*P*(R|**Y**_*i*_,*s*) for *ρ*<1, for each child (in all samples, we identified *ϕ*>1 for *ρ*>1 and *ϕ*<1 for *ρ*<1). We used least-squares regression to test for associations between covariates and children’s log-transformed probability of being in the high-risk group, using estimated regression parameters to measure relative risks (**Table 3**). Models included a setting term to account for differential prevalence of high-risk children. We pooled relative risk estimates across our draws of *θ* to recover their distribution.

### Simulation study

To examine potential bias in conventional estimates of naturally-acquired immune protection, we used our model of the natural history of rotavirus infection to simulate individual histories of infection and RVGE over the first three years of life, sampling from estimated parameters describing the effects of age (*β*_0_, *β*_1_, *β*_2_) and previous infection (*ψ*_1,_ *ψ*_2_, *ψ*_3_) on susceptibility to RVGE and infection, respectively. We conducted simulations under an external force of infection (*Λ*) ranging from 0.2 to 4 infections per year, assigning 0% to 50% (*α*) of children to the high-risk subgroup *R*; values of *ϕ* and *ρ* were drawn independently of *α* so that we could determine the effect of differences in the proportion of high-risk children on estimates of protection. We sampled exponentially-distributed infection times (calculated from time of birth or previous infection), and defined the occurrence of RVGE for each individual infection as a Bernoulli random variable using the model-predicted probability of RVGE given infection.

For each cohort simulation, we measured the hazard ratio for reinfection and RVGE from the incidence rate (*IR*_*k*_) of infection and RVGE after one, two, or three previous infections, relative to the *IR*_0_ from birth. We also measured the relative risk of RVGE given reinfection among children who had experienced one, two, or three previous infections, relative to those with no previous infections, calculated from the proportion (*p*_*k*_) of infections with RVGE. We defined estimates of natural immune efficacy ($\hat{NE}$) as

$${\hat{NE}}_{k}=1-\frac{IR_{k}}{IR_{0}}$$ (11)

for protection against infection and RVGE among children who had experienced *k* previous infections, and

$${\hat{NE}}_{k}=1-\frac{p_{k}}{p_{0}}$$ (12)

for risk of RVGE given infection among children who had experienced *k* previous infections.

## Supporting information

S1 Fig Consistency of parameter estimates under the original and MCMC inference approaches (1 of 2). We illustrate parameter estimates under the original kernel-based approach (histograms) and from each of the 10 Markov chain Monte Carlo chains, overlaying their probability densities and presenting thinned draws from the parameter trace plots over 150,000 iterations (after 50,000 burn-in iterations). Parameters include: (**A**) hazard ratios for infection *ψ*_1_,*ψ*_2_, and *ψ*_3_; (**B** and **C**) setting-specific force of infection for Vellore and Mexico City; and (**D**, **E**, and **F**) the polynomial terms describing age-specific diarrhea risk, given infection.
(TIF)

S2 FigConsistency of parameter estimates under the original and MCMC inference approaches (2 of 2).We illustrate parameter estimates and their joint distributions under the original kernel-based approach (histograms) and from each of the 10 Markov chain Monte Carlo chains (colored lines, as indicated in **S1 Fig**), overlaying their probability densities and presenting thinned draws from the parameter trace plots over 150,000 iterations (after 50,000 burn-in iterations). Parameters include (**A**) hazard ratio for infection *ϕ*, (**B**) relative risk of RVGE given infection *ρ*, (**C**) prevalence of the baseline risk group in Mexico City $\alpha_{M}^{00}$, and (**D**) Vellore $\alpha_{V}^{00}$. (**E**) We plot samples from the joint distribution of $\alpha_{M}^{11}$ and $\alpha_{V}^{11}$, revealing concordance with the original estimates of the joint distribution of *α*_*M*_ and *α*_*V*_ plotted in **Fig 2D**. We also illustrate concordance in samples from the joint distribution of the following parameters under the two approaches: (**F**) $\alpha_{M}^{11}$ and *ϕ*; (**G**) $\alpha_{V}^{11}$ and *ϕ*; (**H**) $\alpha_{M}^{11}$ and *ρ*; and (**I**) $\alpha_{V}^{11}$ and *ρ*.(TIF)

S3 FigMCMC samples for risk groups.We present samples of the proportion of individuals belonging to the various risk groups from each of the 10 Markov chain Monte Carlo chains, overlaying their posterior distributions and presenting thinned draws from the parameter trace plots over 150,000 iterations (after 50,000 burn-in iterations). The first panels indicate the proportion of individuals belonging to the risk group with modified rates of acquiring infection (i.e., for whom *ϕ* applies) in (**A**) Vellore and (**B**) Mexico City, while the next two panels (**C** and **D**) illustrate the proportion of these individuals with modified risk of diarrhea given infection (i.e., for whom *ρ* applies). Among the proportion without modified rates of acquiring infection (i.e., for whom *ϕ* does not apply), the proportion who also do not experience modified risk of diarrhea given infection (i.e., for whom *ρ* does not apply) is illustrated in the final two panels (**E** and **F**). Convergence of the parameters $\alpha_{M}^{01},\;\alpha_{V}^{01},\;\alpha_{M}^{10}$, and $\alpha_{V}^{10}$ to zero (see **S3 Table**) results in the concentration of probability mass very close to one across all chains (panels **C**–**F**).(TIF)

S1 Table Testing for linkages in susceptibility to infection and disease under Markov Chain Monte Carlo sampling. We present parameter estimates and their 95% credible intervals under the model including strata with distinct susceptibility to infection and disease (either or in combination), as well as posterior probabilities for hypothesis tests.
(DOCX)

S2 Table Study design, enrollment, and follow-up. ^1^Sample restricted to children who completed 3 years of follow-up (83% of initial cohort of 452 children) ^2^The total number was not provided in the original study, but was calculated from the information that the 963 tested episodes represented 85% of the total reported episodes. ^2^In the Mexico City cohort, the mean age of asymptomatic infections detected by shedding is lower than the mean age of asymptomatic infections detected by seroconversion alone (*p*<0.0001). ^3^In the Vellore cohort, the mean age of asymptomatic infections detected by shedding is lower than the mean age of asymptomatic infections detected by seroconversion alone (*p*<0.001).
(DOCX)

S3 Table Primary estimates of naturally-acquired immune protection. ^1^Incidence is measured per 100 child-months at risk ^2^The original studies applied differing definitions for moderate-to-severe RVGE; here we consider episodes with Vesikari score ≥11 to constitute moderate-to-severe RVGE.
(DOCX)

S1 Text Supplemental materials. Text including supplemental methods, tables, and figures.
(PDF)
