# Supplementary figures and images for: Heterogeneous susceptibility to rotavirus infection and gastroenteritis in two birth cohort studies: Parameter estimation and epidemiological implications
Source: PLoS Comput Biol. 2019 Jul 26;15(7):e1007014. doi: 10.1371/journal.pcbi.1007014 (PMC6690553; doi:10.1371/journal.pcbi.1007014)

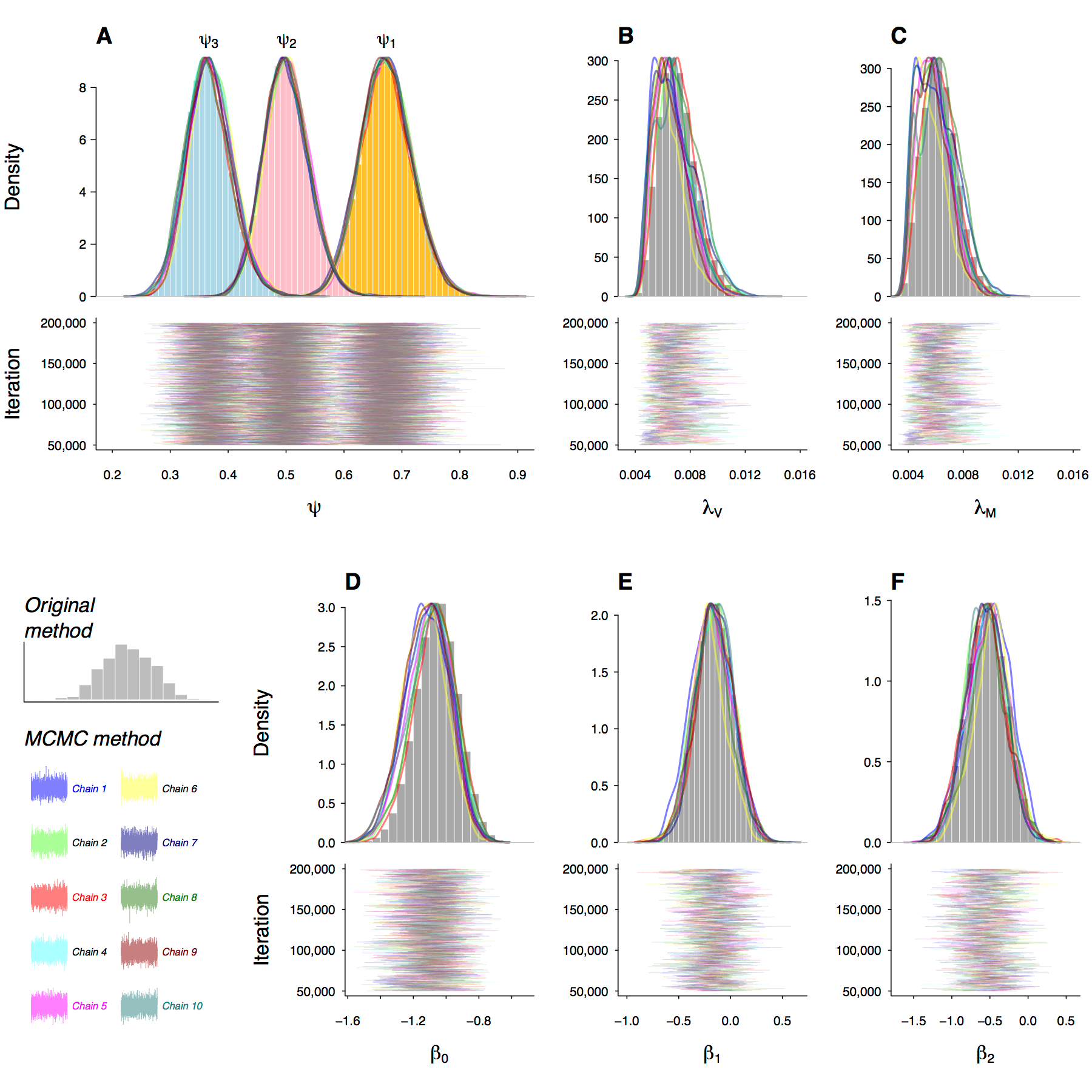

Supplement: S1 Fig — We illustrate parameter estimates under the original kernel-based approach (histograms) and from each of the 10 Markov chain Monte Carlo chains, overlaying their probability densities and presenting thinned draws from the parameter trace plots over 150,000 iterations (after 50,000 burn-in iterations). Parameters include: (A) hazard ratios for infection ψ1,ψ2, and ψ3; (B and C) setting-specific force of infection for Vellore and Mexico City; and (D, E, and F) the polynomial terms describing age-specific diarrhea risk, given infection. (TIF) [file pcbi.1007014.s001.tif]

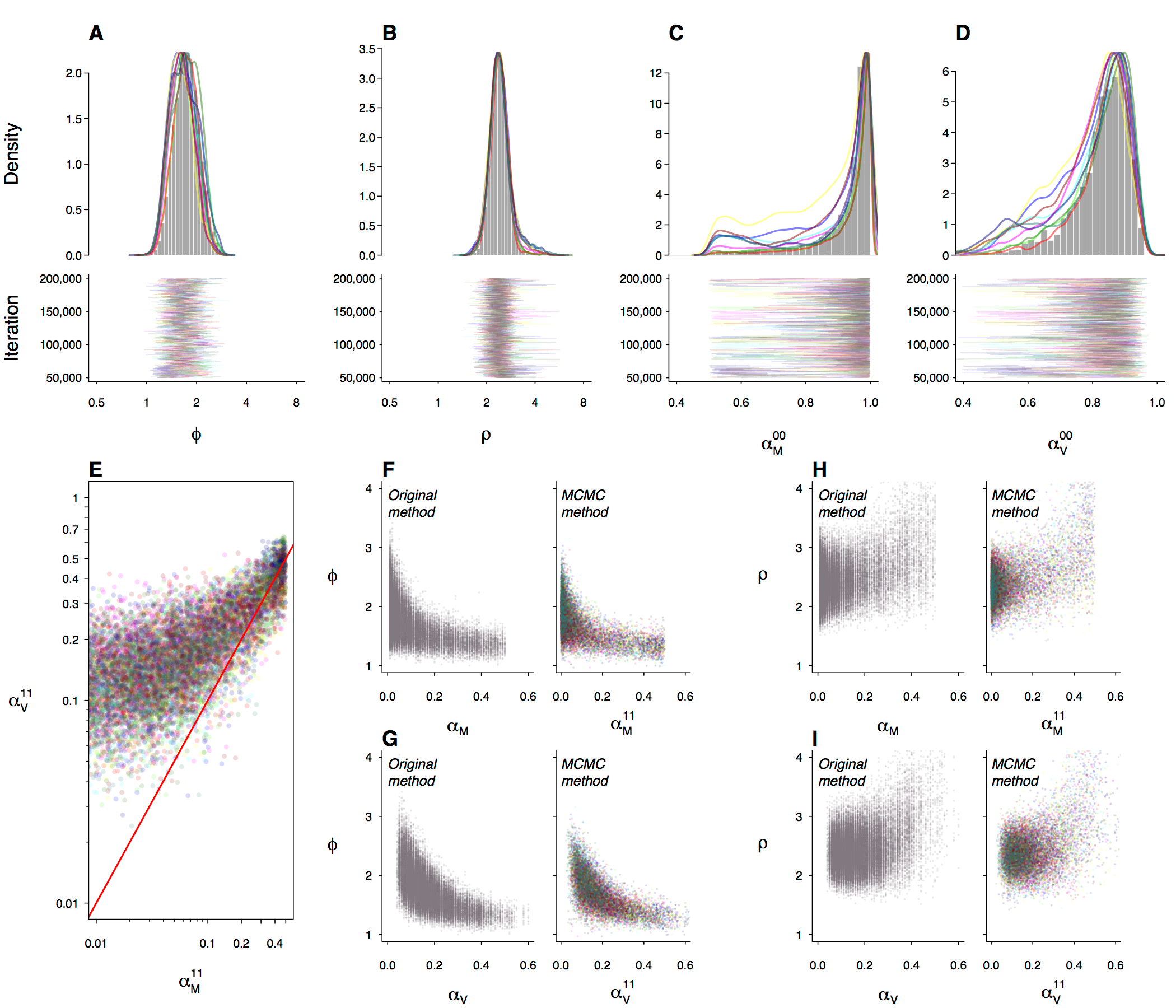

Supplement: S2 Fig — We illustrate parameter estimates and their joint distributions under the original kernel-based approach (histograms) and from each of the 10 Markov chain Monte Carlo chains (colored lines, as indicated in S1 Fig), overlaying their probability densities and presenting thinned draws from the parameter trace plots over 150,000 iterations (after 50,000 burn-in iterations). Parameters include (A) hazard ratio for infection ϕ, (B) relative risk of RVGE given infection ρ, (C) prevalence of the baseline risk group in Mexico City αM00, and (D) Vellore αV00. (E) We plot samples from the joint distribution of αM11 and αV11, revealing concordance with the original estimates of the joint distribution of αM and αV plotted in Fig 2D. We also illustrate concordance in samples from the joint distribution of the following parameters under the two approaches: (F) αM11 and ϕ; (G) αV11 and ϕ; (H) αM11 and ρ; and (I) αV11 and ρ. (TIF) [file pcbi.1007014.s002.tif]

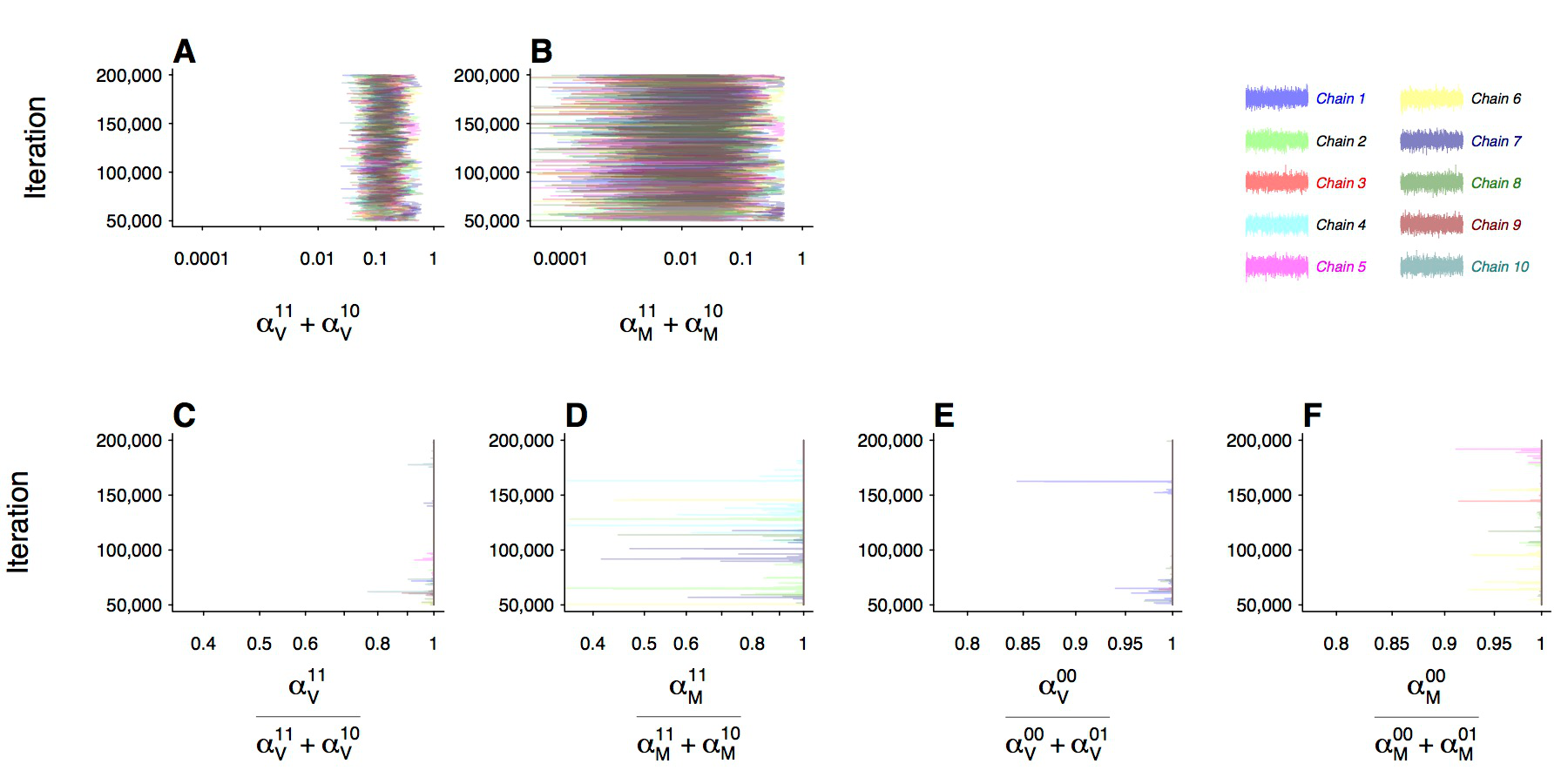

Supplement: S3 Fig — We present samples of the proportion of individuals belonging to the various risk groups from each of the 10 Markov chain Monte Carlo chains, overlaying their posterior distributions and presenting thinned draws from the parameter trace plots over 150,000 iterations (after 50,000 burn-in iterations). The first panels indicate the proportion of individuals belonging to the risk group with modified rates of acquiring infection (i.e., for whom ϕ applies) in (A) Vellore and (B) Mexico City, while the next two panels (C and D) illustrate the proportion of these individuals with modified risk of diarrhea given infection (i.e., for whom ρ applies). Among the proportion without modified rates of acquiring infection (i.e., for whom ϕ does not apply), the proportion who also do not experience modified risk of diarrhea given infection (i.e., for whom ρ does not apply) is illustrated in the final two panels (E and F). Convergence of the parameters αM01,αV01,αM10, and αV10 to zero (see S3 Table) results in the concentration of probability mass very close to one across all chains (panels C–F). (TIF) [file pcbi.1007014.s003.tif]
