# Supplementary material for: Heterogeneous susceptibility to rotavirus infection and gastroenteritis in two birth cohort studies: Parameter estimation and epidemiological implications
Source: PLoS Comput Biol. 2019 Jul 26;15(7):e1007014. doi: 10.1371/journal.pcbi.1007014 (PMC6690553; doi:10.1371/journal.pcbi.1007014)
Supplement: S1 Table — We present parameter estimates and their 95% credible intervals under the model including strata with distinct susceptibility to infection and disease (either or in combination), as well as posterior probabilities for hypothesis tests. (DOCX) [file pcbi.1007014.s004.docx]

**S1 Table. Testing for linkages in susceptibility to infection and disease under Markov Chain Monte Carlo sampling.**

| **Setting** | **Parameter or test** | **Estimate (95% CrI)** | **Posterior probability** |
| --- | --- | --- | --- |
| Mexico City |  |  |  |
|  | $\alpha_{M}^{00}$ | 0.956 (0.535, 0.999) |  |
|  | $\alpha_{M}^{11}$ | 0.044 (6.94×10^–4^, 0.465) |  |
|  | $\alpha_{M}^{10}$ | 3.23×10^–27^ (3.41×10^–95^, 2.36×10^–4^) |  |
|  | $\alpha_{M}^{01}$ | 7.58×10^–29^ (1.50×10^–141^, 2.06×10^–4^) |  |
|  | $\Pr\left[ \frac{\alpha_{M}^{11}}{\alpha_{M}^{01}+\alpha_{M}^{11}}<\frac{\alpha_{M}^{10}}{\alpha_{M}^{10}+\alpha_{M}^{00}} \right]$ |  | <6.7×10^–6^ |
|  | $\Pr\left[ \frac{\alpha_{M}^{11}}{\alpha_{M}^{01}+\alpha_{M}^{11}}<\frac{\alpha_{M}^{10}}{\alpha_{M}^{10}+\alpha_{M}^{00}} \right]$ |  | <6.7×10^–6^ |
| Vellore |  |  |  |
|  | $\alpha_{V}^{00}$ | 0.836 (0.520, 0.941) |  |
|  | $\alpha_{V}^{11}$ | 0.164 (0.059, 0.480) |  |
|  | $\alpha_{V}^{10}$ | 5.29×10^–27^ (1.40×10^–99^, 9.92×10^–5^) |  |
|  | $\alpha_{V}^{01}$ | 1.15×10^–41^ (1.07×10^–98^, 1.30×10^–4^) |  |
|  | $\Pr\left[ \frac{\alpha_{V}^{11}}{\alpha_{V}^{01}+\alpha_{V}^{11}}<\frac{\alpha_{V}^{10}}{\alpha_{V}^{10}+\alpha_{V}^{00}} \right]$ |  | <6.7×10^–6^ |
|  | $\Pr\left[ \frac{\alpha_{V}^{11}}{\alpha_{V}^{01}+\alpha_{V}^{11}}<\frac{\alpha_{V}^{10}}{\alpha_{V}^{10}+\alpha_{V}^{00}} \right]$ |  | <6.7×10^–6^ |
