# Supplementary material for: Heterogeneous susceptibility to rotavirus infection and gastroenteritis in two birth cohort studies: Parameter estimation and epidemiological implications
Source: PLoS Comput Biol. 2019 Jul 26;15(7):e1007014. doi: 10.1371/journal.pcbi.1007014 (PMC6690553; doi:10.1371/journal.pcbi.1007014)
Supplement: S2 Table — 1Sample restricted to children who completed 3 years of follow-up (83% of initial cohort of 452 children) 2The total number was not provided in the original study, but was calculated from the information that the 963 tested episodes represented 85% of the total reported episodes. 2In the Mexico City cohort, the mean age of asymptomatic infections detected by shedding is lower than the mean age of asymptomatic infections detected by seroconversion alone (p<0.0001). 3In the Vellore cohort, the mean age of asymptomatic infections detected by shedding is lower than the mean age of asymptomatic infections detected by seroconversion alone (p<0.001). (DOCX) [file pcbi.1007014.s005.docx]

**S2 Table. Study design, enrollment, and follow-up.**

|  | **Mexico City cohort** | **Vellore cohort** |
| --- | --- | --- |
| Duration of follow-up | 24 months | 36 months |
| Frequency of asymptomatic stool testing | Weekly | Every 2 weeks |
| Frequency of serological testing | Every 4 months | At least every 6 months |
| Definition of rotavirus shedding | ELISA positive | 2x ELISA positive or RT-PCR positive |
| Definition of seroconversion | 4-fold rise in IgG or IgA | 4-fold rise in IgG or 3-fold rise in IgA |
| Study population | 200 children | 373 children^1^ |
| Child-months of observation | 3699/4800 (77%) | 13341/13428 (99%) |
| Asymptomatic stool samples tested | 15503 | 26902 |
| Of all scheduled tests | 15503/20800 (75%) | 26902/29094 (92%) |
| Of all scheduled tests while child was retained in follow-up | 15503/16029 (97%) | 26902/28906 (93%) |
| Diarrheal episodes tested, of all reported diarrheal episodes | 963/1133^2^ (85%) | 1829/1856 (99%) |
| Serum samples tested, of all scheduled tests | 1037/1080 (96%) | 2565/2598 (99%) |
| Infections detected | 316 | 1103 |
| From diarrheal episodes | 89/316 (28%) | 282/1103 (26%) |
| From asymptomatic shedding | 88/316 (28%) | 237/1103 (21%) |
| From seroconversion only | 139/316 (44%) | 584/1103 (53%) |
| Mean age (d), asymptomatic infections detected by seroconversion only | 445 (SD=167)^3^ | 626 (SD=308)^4^ |
| Mean age (d), asymptomatic infections detected by shedding | 339 (SD=187)^3^ | 542 (SD=342)^4^ |
| Rotavirus-negative diarrhea samples | 874/963 (91%) | 1547/1829 (85%) |

^1^Sample restricted to children who completed 3 years of follow-up (83% of initial cohort of 452 children)

^2^The total number was not provided in the original study, but was calculated from the information that the 963 tested episodes represented 85% of the total reported episodes.

^2^In the Mexico City cohort, the mean age of asymptomatic infections detected by shedding is lower than the mean age of asymptomatic infections detected by seroconversion alone (*p*<0.0001).

^3^In the Vellore cohort, the mean age of asymptomatic infections detected by shedding is lower than the mean age of asymptomatic infections detected by seroconversion alone (*p*<0.001).
