# Supplementary material for: Heterogeneous susceptibility to rotavirus infection and gastroenteritis in two birth cohort studies: Parameter estimation and epidemiological implications
Source: PLoS Comput Biol. 2019 Jul 26;15(7):e1007014. doi: 10.1371/journal.pcbi.1007014 (PMC6690553; doi:10.1371/journal.pcbi.1007014)
Supplement: S3 Table — 1Incidence is measured per 100 child-months at risk 2The original studies applied differing definitions for moderate-to-severe RVGE; here we consider episodes with Vesikari score ≥11 to constitute moderate-to-severe RVGE. (DOCX) [file pcbi.1007014.s006.docx]

**S3 Table. Primary estimates of naturally-acquired immune protection.**

| **Outcome** | **Previous infections** | **Mexico City cohort** | | | **Vellore cohort** | | |
| --- | --- | --- | --- | --- | --- | --- | --- |
|  |  | **Infections** | **Incidence^1^** | **Est. protection, % (95% CI)** | **Infections** | **Incidence^1^** | **Est. protection, % (95% CI)** |
| Infection |  |  |  |  |  |  |  |
|  | 0 | 164 | 11.3 | ref. | 371 | 13.8 | ref. |
|  | 1 | 102 | 8.3 | 38 (17, 50) | 338 | 8.5 | 39 (29, 47) |
|  | 2 | 40 | 5.4 | 60 (41,72) | 236 | 6.7 | 52 (43, 59) |
|  | 3 | 9 | 4.2 | 66 (33, 83) | 100 | 4.7 | 67 (59, 74) |
| Any RVGE |  |  |  |  |  |  |  |
|  | 0 | 64 | 4.4 | ref. | 111 | 4.1 | ref. |
|  | 1 | 16 | 1.3 | 77 (60, 88) | 95 | 2.4 | 43 (24, 56) |
|  | 2 | 8 | 1.1 | 83 (64, 92) | 43 | 1.2 | 71 (59, 80) |
|  | 3 | 1 | 0.5 | 92 (44, 99) | 18 | 0.8 | 81 (69, 88) |
| Moderate-to-severe RVGE^2^ |  |  |  |  |  |  |  |
|  | 0 | 12 | 0.8 | ref. | 27 | 1.0 | ref. |
|  | 1 | 2 | 0.2 | 82 (38, 100) | 25 | 0.6 | 18 (–57, 57) |
|  | 2 | 0 | 0 | 100 | 11 | 0.3 | 57 (6, 80) |
|  | 3 | 0 | 0 | 100 | 3 | 0.1 | 79 (29, 94) |

^1^Incidence is measured per 100 child-months at risk

^2^The original studies applied differing definitions for moderate-to-severe RVGE; here we consider episodes with Vesikari score ≥11 to constitute moderate-to-severe RVGE.
