# Supplementary material for: Heterogeneous susceptibility to rotavirus infection and gastroenteritis in two birth cohort studies: Parameter estimation and epidemiological implications
Source: PLoS Comput Biol. 2019 Jul 26;15(7):e1007014. doi: 10.1371/journal.pcbi.1007014 (PMC6690553; doi:10.1371/journal.pcbi.1007014)
Supplement: S1 Text — Text including supplemental methods, tables, and figures. (PDF) [file pcbi.1007014.s007.pdf]

S1 Text: Inference via Markov chain Monte Carlo sampling.

To verify that the semiparametric approach described in the main text yielded unbiased estimates of the risk groups and susceptibility parameters, we also sampled parameters via Markov chain Monte Carlo (MCMC) sampling according to the likelihoods defined in the main text. This approach further allowed us to consider distinct risk groups for infection and disease (RVGE) susceptibility within each cohort—a model specification that could not be addressed efficiently under the original semiparametric approach.

Here, we defined the population of each cohort as being partitioned into four (rather than two) strata:

1. Individuals with unmodified risk of infection and disease, constituting a proportion $P(R^{00}) = \alpha_s^{00}$ of the population of setting *s*;
2. Individuals with hazard ratio ϕ of acquiring infection, and relative risk ρ of disease given infection, constituting a proportion $P(R^{11}) = \alpha_s^{11}$ of the population of setting *s*;
3. Individuals with hazard ratio ϕ of acquiring infection, and unmodified risk of disease given infection, constituting a proportion $P(R^{10}) = \alpha_s^{10}$ of the population of setting *s*;
4. Individuals with unmodified risk of infection but relative risk ρ of disease given infection, constituting a proportion $P(R^{01}) = \alpha_s^{01}$ of the population of setting *s*.

Here, we took $\alpha_s^{00} + \alpha_s^{11} + \alpha_s^{10} + \alpha_s^{01} = 1$, and obtained the likelihood contribution for each child *i*, $H_s(i)$, via the total probability,

$$H_s(i) = \sum_k \alpha_s^k \prod_j L(i,j|R^k, s).$$

To distinguish the risk groups, we again assigned the definition $\alpha_M^{11} \sim \mathrm{Unif}(0,0.5)$ and estimated the parameters ϕ and ρ (as well as risk strata prevalences) conditioned on this particular stratum covering a minority of children in Mexico City; as estimates of ϕ and ρ were not subject to priors or constraints, the stratum α_M^{11} could be defined to include either higher- or lower-risk children in terms of susceptibility to infection and disease. To sample parameters from the posterior distribution, we ran ten chains in parallel for 200,000 iterations each. We updated the state of the chains via a Metropolis-Hastings procedure, sampling proposed candidate values based on random normal draws centered at the current values on a log scale. We discarded the initial 50,000 iterations from each chain as burn-in and subsequently saved the state of the chain at every 10th iteration.

We tested for linkage between the traits of susceptibility to infection and susceptibility to disease given infection by comparing the probabilities that an individual would experience enhanced risk for infection and for disease, given the presence or absence of enhanced susceptibility to the other entity. Under the condition that $\phi > 1$ and $\phi > 1$, such that R^{11} indicates the population with enhanced susceptibility to both infection and disease, the associated tests were

$$\Pr\left[\frac{\alpha_s^{11}}{\alpha_s^{01} + \alpha_s^{11}} = \frac{\alpha_s^{10}}{\alpha_s^{10} + \alpha_s^{00}}\right]$$

for the relative probability of enhanced susceptibility to infection given enhanced susceptibility to disease, and

$$\Pr\left[\frac{\alpha_s^{11}}{\alpha_s^{10} + \alpha_s^{11}} = \frac{\alpha_s^{01}}{\alpha_s^{01} + \alpha_s^{00}}\right]$$

for the relative probability of enhanced susceptibility to disease given enhanced susceptibility to infection. We identified strong support for both conditions in our estimates (**S1 Table**). In addition, we identified near-identical estimates of the other parameters (which are common to both models) under the two approaches (**S1 Fig**, **S2 Fig**, **S3 Fig**). As our estimates of α_M^{10}, α_M^{01}, α_V^{10}, and α_V^{01} converge to zero (**S1 Table**, **S3 Fig**), the "full" model considered here collapses to that considered in the original analysis.

Table S1. Testing for linkages in susceptibility to infection and disease under Markov Chain Monte Carlo sampling.

Setting	Parameter or test	Estimate (95% CrI)	Posterior probability
Mexico City			
	α_M^{00}	0.956 (0.535, 0.999)	
	α_M^{11}	0.044 (6.94×10^{-4}, 0.465)	
	α_M^{10}	3.23×10^{-27} (3.41×10^{-95}, 2.36×10^{-4})	
	α_M^{01}	7.58×10^{-29} (1.50×10^{-141}, 2.06×10^{-4})	
	$\mathrm{Pr}\left[\frac{\alpha_M^{11}}{\alpha_M^{01}+\alpha_M^{11}} < \frac{\alpha_M^{10}}{\alpha_M^{10}+\alpha_M^{00}}\right]$		<6.7×10^{-6}
	$\mathrm{Pr}\left[\frac{\alpha_M^{11}}{\alpha_M^{01}+\alpha_M^{11}} < \frac{\alpha_M^{10}}{\alpha_M^{10}+\alpha_M^{00}}\right]$		<6.7×10^{-6}
Vellore			
	α_V^{00}	0.836 (0.520, 0.941)	
	α_V^{11}	0.164 (0.059, 0.480)	
	α_V^{10}	5.29×10^{-27} (1.40×10^{-99}, 9.92×10^{-5})	
	α_V^{01}	1.15×10^{-41} (1.07×10^{-98}, 1.30×10^{-4})	
	$\mathrm{Pr}\left[\frac{\alpha_V^{11}}{\alpha_V^{01}+\alpha_V^{11}} < \frac{\alpha_V^{10}}{\alpha_V^{10}+\alpha_V^{00}}\right]$		<6.7×10^{-6}
	$\mathrm{Pr}\left[\frac{\alpha_V^{11}}{\alpha_V^{01}+\alpha_V^{11}} < \frac{\alpha_V^{10}}{\alpha_V^{10}+\alpha_V^{00}}\right]$		<6.7×10^{-6}

Table S2. Study design, enrollment, and follow-up.

	Mexico City cohort	Vellore cohort
Duration of follow-up	24 months	36 months
Frequency of asymptomatic stool testing	Weekly	Every 2 weeks
Frequency of serological testing	Every 4 months	At least every 6 months
Definition of rotavirus shedding	ELISA positive	2x ELISA positive or RT-PCR positive
Definition of seroconversion	4-fold rise in IgG or IgA	4-fold rise in IgG or 3-fold rise in IgA
Study population	200 children	373 children[1]
Child-months of observation	3699/4800 (77%)	13341/13428 (99%)
Asymptomatic stool samples tested	15503	26902
Of all scheduled tests	15503/20800 (75%)	26902/29094 (92%)
Of all scheduled tests while child was retained in follow-up	15503/16029 (97%)	26902/28906 (93%)
Diarrheal episodes tested, of all reported diarrheal episodes	963/1133[2] (85%)	1829/1856 (99%)
Serum samples tested, of all scheduled tests	1037/1080 (96%)	2565/2598 (99%)
Infections detected	316	1103
From diarrheal episodes	89/316 (28%)	282/1103 (26%)
From asymptomatic shedding	88/316 (28%)	237/1103 (21%)
From seroconversion only	139/316 (44%)	584/1103 (53%)
Mean age (d), asymptomatic infections detected by seroconversion only	445 (SD=167)[3]	626 (SD=308)[4]
Mean age (d), asymptomatic infections detected by shedding	339 (SD=187)[3]	542 (SD=342)[4]
Rotavirus-negative diarrhea samples	874/963 (91%)	1547/1829 (85%)

[1]Sample restricted to children who completed 3 years of follow-up (83% of initial cohort of 452 children)
[2]The total number was not provided in the original study, but was calculated from the information that the 963 tested episodes represented 85% of the total reported episodes.
[2]In the Mexico City cohort, the mean age of asymptomatic infections detected by shedding is lower than the mean age of asymptomatic infections detected by seroconversion alone (p<0.0001).
[3]In the Vellore cohort, the mean age of asymptomatic infections detected by shedding is lower than the mean age of asymptomatic infections detected by seroconversion alone (p<0.001).

Table S3. Primary estimates of naturally-acquired immune protection.

Outcome	Previous infections	Mexico City cohort			Vellore cohort		
		Infections	Incidence[1]	Est. protection, % (95% CI)	Infections	Incidence[1]	Est. protection, % (95% CI)
Infection							
	0	164	11.3	ref.	371	13.8	ref.
	1	102	8.3	38 (17, 50)	338	8.5	39 (29, 47)
	2	40	5.4	60 (41,72)	236	6.7	52 (43, 59)
	3	9	4.2	66 (33, 83)	100	4.7	67 (59, 74)
Any RVGE							
	0	64	4.4	ref.	111	4.1	ref.
	1	16	1.3	77 (60, 88)	95	2.4	43 (24, 56)
	2	8	1.1	83 (64, 92)	43	1.2	71 (59, 80)
	3	1	0.5	92 (44, 99)	18	0.8	81 (69, 88)
Moderate-to-severe RVGE[2]							
	0	12	0.8	ref.	27	1.0	ref.
	1	2	0.2	82 (38, 100)	25	0.6	18 (–57, 57)
	2	0	0	100	11	0.3	57 (6, 80)
	3	0	0	100	3	0.1	79 (29, 94)

[1]Incidence is measured per 100 child-months at risk

[2]The original studies applied differing definitions for moderate-to-severe RVGE; here we consider episodes with Vesikari score ≥11 to constitute moderate-to-severe RVGE.

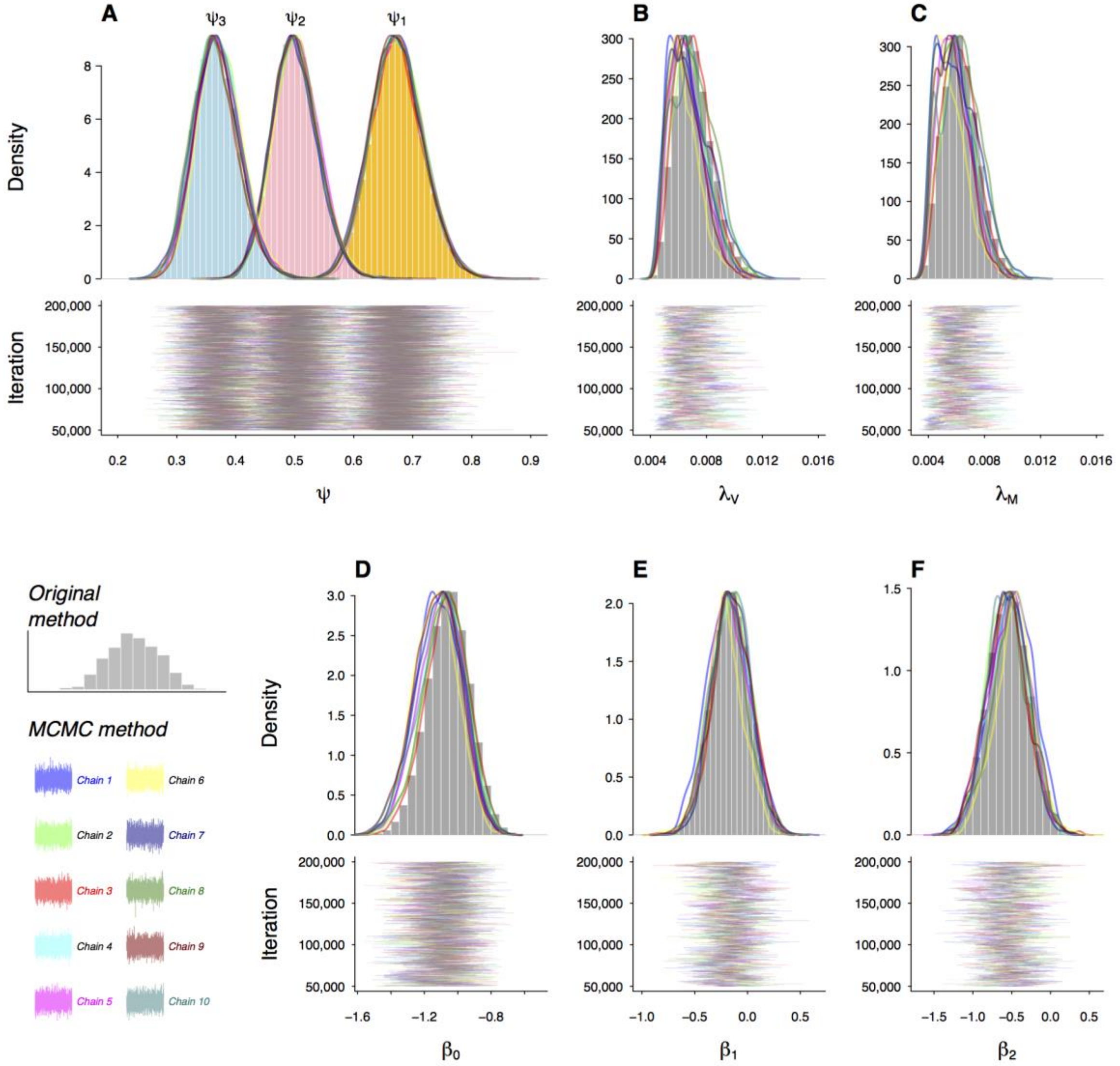

Figure S1: Consistency of parameter estimates under the original and MCMC inference approaches (1 of 2). We illustrate parameter estimates under the original kernel-based approach (histograms) and from each of the 10 Markov chain Monte Carlo chains, overlaying their probability densities and presenting thinned draws from the parameter trace plots over 150,000 iterations (after 50,000 burn-in iterations). Parameters include: (**A**) hazard ratios for infection $\psi_1, \psi_2, \text{and } \psi_3$; (**B** and **C**) setting-specific force of infection for Vellore and Mexico City; and (**D**, **E**, and **F**) the polynomial terms describing age-specific diarrhea risk, given infection.

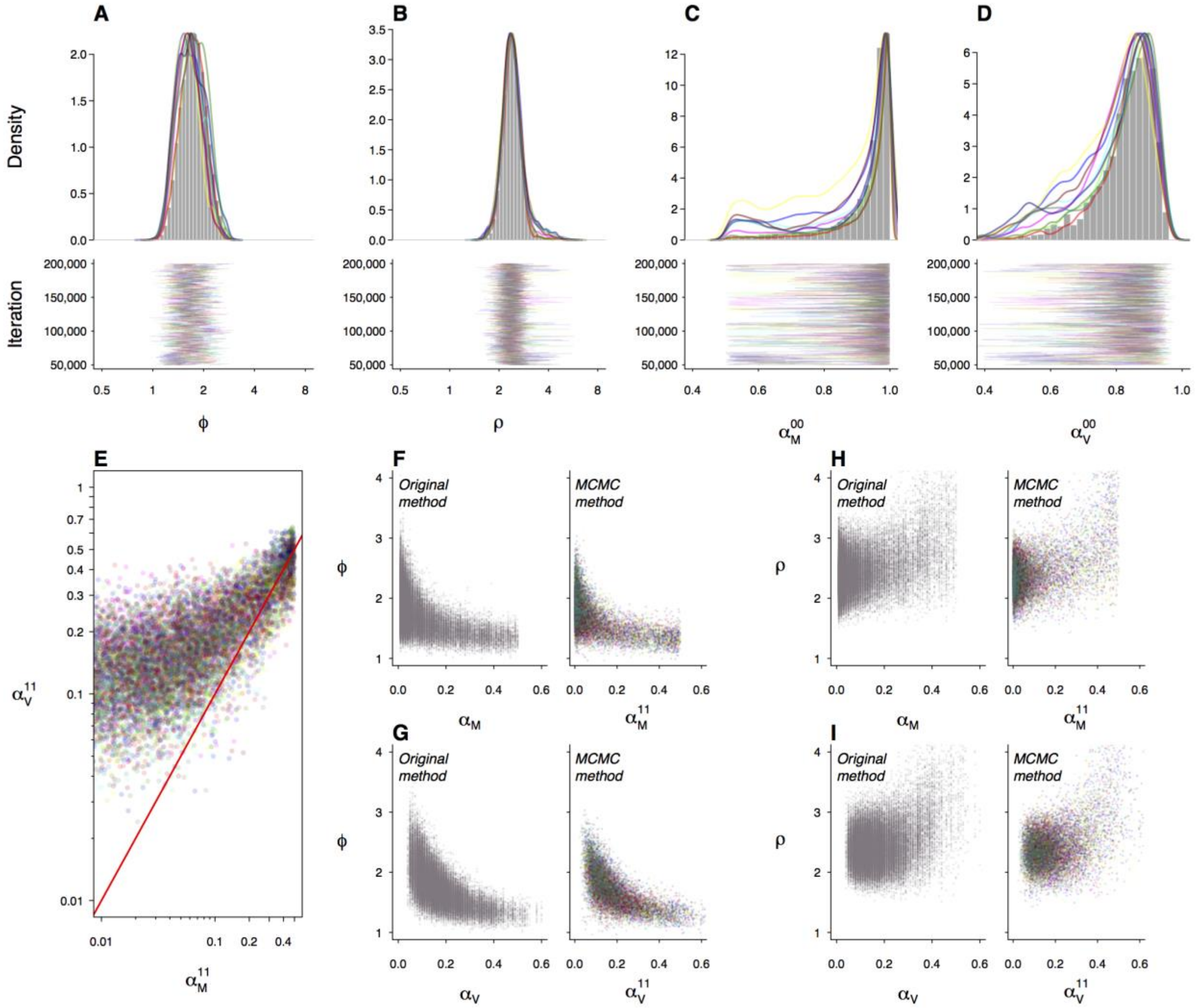

Figure S2: Consistency of parameter estimates under the original and MCMC inference approaches (2 of 2). We illustrate parameter estimates and their joint distributions under the original kernel-based approach (histograms) and from each of the 10 Markov chain Monte Carlo chains (colored lines, as indicated in **S1 Fig**), overlaying their probability densities and presenting thinned draws from the parameter trace plots over 150,000 iterations (after 50,000 burn-in iterations). Parameters include (**A**) hazard ratio for infection ϕ, (**B**) relative risk of RVGE given infection ρ, (**C**) prevalence of the baseline risk group in Mexico City α_M^{00}, and (**D**) Vellore α_V^{00}. (**E**) We plot samples from the joint distribution of α_M^{11} and α_V^{11}, revealing concordance with the original estimates of the joint distribution of α_M and α_V plotted in **Figure 2D**. We also illustrate concordance in samples from the joint distribution of the following parameters under the two approaches: (**F**) α_M^{11} and ϕ; (**G**) α_V^{11} and ϕ; (**H**) α_M^{11} and ρ; and (**I**) α_V^{11} and ρ.

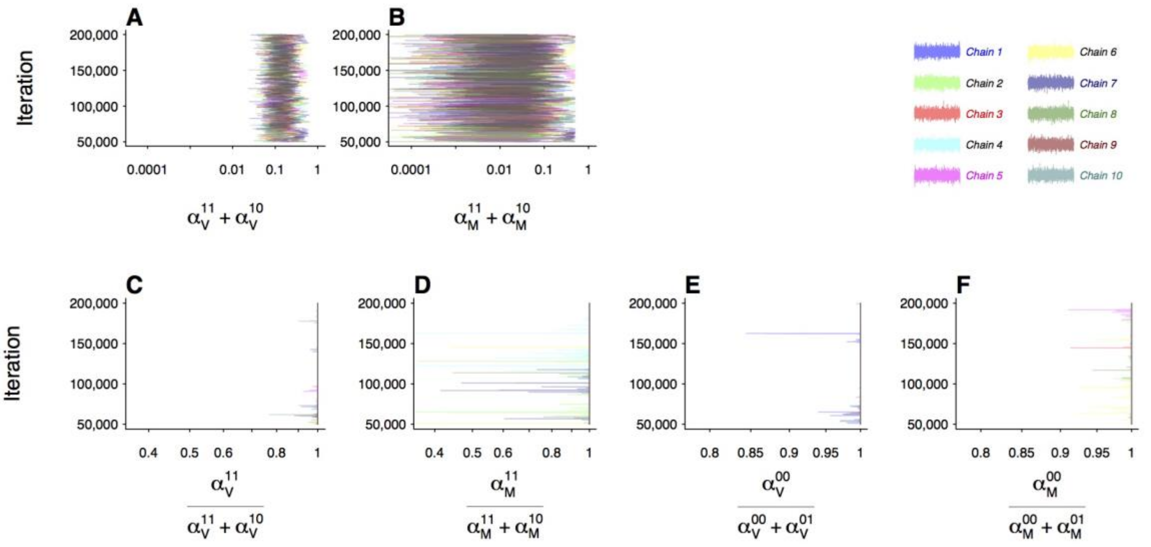

Figure S3: MCMC samples for risk groups. We present samples of the proportion of individuals belonging to the various risk groups from each of the 10 Markov chain Monte Carlo chains, overlaying their posterior distributions and presenting thinned draws from the parameter trace plots over 150,000 iterations (after 50,000 burn-in iterations). The first panels indicate the proportion of individuals belonging to the risk group with modified rates of acquiring infection (i.e., for whom ϕ applies) in (**A**) Vellore and (**B**) Mexico City, while the next two panels (**C** and **D**) illustrate the proportion of these individuals with modified risk of diarrhea given infection (i.e., for whom ρ applies). Among the proportion without modified rates of acquiring infection (i.e., for whom ϕ does not apply), the proportion who also do not experience modified risk of diarrhea given infection (i.e., for whom ρ does not apply) is illustrated in the final two panels (**E** and **F**). Convergence of the parameters α_M^{01}, α_V^{10}, α_M^{10}, and α_V^{10} to zero (see **Table S3**) results in the concentration of probability mass very close to one across all chains (panels **C**–**F**).
